# Photobiomodulation on vocal training and rehabilitation: Delphi consensus based on experts

**DOI:** 10.1590/2317-1782/e20230356en

**Published:** 2025-02-05

**Authors:** Emerson Soares Pontes, Thays Garcia Vaiano, Roberto Sávio de Assunção Bastos, João Marcos da Trindade Duarte, Émile Rocha Santana, Leonardo Wanderley Lopes

**Affiliations:** 1 Universidade Federal da Paraíba – UFPB - João Pessoa (PB), Brasil.; 2 Centro de Estudos da Voz – CEV - São Paulo (SP), Brasil.; 3 Hospital Pronto Socorro Municipal Mário Pinotto - Belém (PA), Brasil.; 4 Universidade do Estado da Bahia – UNEB - Salvador (BA), Brasil.

**Keywords:** Voice, Voice Quality, Voice Training, Voice Disorders, Low-level Light Therapy, Lasers, Delphi Technique

## Abstract

**Purpose:**

To develop a consensus among speech-language pathologists who are voice specialists regarding the criteria for recommending and using photobiomodulation in the context of vocal therapy and training.

**Methods:**

Seven speech-language pathologists, experts in voice, and with experience in using photobiomodulation in vocal therapy and training participated. The Delphi technique was used to achieve consensus from a panel of experts accessed independently in two phases of collection. In Phase 1, the experts were contacted individually and participated in an interview with 12 questions to gather opinions on the use of photobiomodulation in the investigated context. The experts' responses were used to construct a questionnaire with 55 items presented as statements. The experts were asked to analyze each item and indicate their level of agreement on a five-point Likert scale. The content validity coefficient (CVC) was used to investigate the degree of agreement among the judges and to select the final items of the consensus.

**Results:**

Consensus was reached among the experts on 34 items investigated in this study, with a CVC ≥ 0.75. It was observed that 31 items achieved an excellent CVC (≥ 0.78), 14 items with a good CVC (0.60 ≥ CVC ≤ 0.77) and 10 items with a poor CVC (≤ 0.59). The total CVC was considered excellent, with a value of 0.78.

**Conclusion:**

There was a consensus among experts about the use of photobiomodulation in vocal habilitation and rehabilitation. It has the potential to improve the criteria for prescribing and using this device by speech-language pathologists. The findings may be useful to improve the criteria for prescribing and the use of this device by speech-language pathologists, in addition to subsidizing the development of future research and clinical recommendations in the area.

## INTRODUCTION

In general, speech-language pathology intervention in voice can include rehabilitation for dysphonia or training for the enhancement and conditioning of spoken and sung voice. In the context of rehabilitation, vocal therapy is considered the treatment of choice, especially in cases where vocal behavior is clearly involved as an etiological, aggravating, or maintaining factor of dysphonia^(1-8)^. Vocal therapy, conducted by speech-language pathologists, aims to modify respiratory, phonatory, and resonant adjustments and promote changes in inefficient vocal behaviors learned throughout life. Overall, vocal therapy involves guidance on vocal health, awareness of vocal psychodynamics, and vocal training with exercises and devices to modify the motor adjustments involved in voice production^(1-9)^. In summary, all strategies used in vocal therapy aim to develop the best possible voice for the patient's/client's needs.

In this context, speech-language pathologists may use devices to facilitate their clinical interventions for modifying therapeutic targets and optimizing the rehabilitation or habilitation process^(10)^. These devices can be classified as volitional when they require an active action or participation from the patient, and non-volitional when they do not require any specific action or behavior from the patient^(11)^. Photobiomodulation (PBM) is classified as a non-volitional device, as its use generally does not require any specific phonatory task along with its application.

PBM is a non-invasive procedure that promotes biostimulation of the irradiated area due to the interaction of visible red and infrared light absorbed by endogenous chromophores. The absorbed light triggers biological reactions at the mitochondrial level, promoting photophysical and photochemical events in biological tissue^(12)^.

In general, PBM has been used to promote tissue repair, modulate inflammatory processes, or produce analgesia in the irradiated region^(13,14)^. In the field of voice, PBM is a complementary strategy in the rehabilitation of dysphonic patients or in the training/conditioning of professional voices^(15-17)^.

In vocal conditioning, whether for spoken or sung voice, one of the primary objectives of vocal training is to improve the individual’s performance according to the required demand. This involves enhancing muscular performance and reducing symptoms related to vocal fatigue^(18)^. The use of voice under adverse conditions, with the recruitment of dysfunctional adjustments in the vocal production system, combined with factors such as prolonged use and high intensity, can lead to vocal fatigue. This condition is generally characterized by reduced phonatory efficiency, associated with vocal hyperfunction and self-reported increased sensation of effort in response to a specific vocal demand^(19-21)^.

The application of PBM in individuals with voice disorders is based on the clinical reasoning that most phonotraumatic lesions involve edematous changes and inflammatory processes in the vocal folds. PBM irradiation can act as a modulator of the inflammatory process by influencing factors such as increased local microcirculation^(17)^; increased ATP synthesis^(22)^; promotion of angiogenesis and vasodilation^(16)^; inhibition of inflammatory mediators such as prostaglandin and cyclooxygenase; reduction of reactive oxygen species and pro-inflammatory cytokines^(23)^; stimulation of fibroblast collagen synthesis, migration capacity, and improvement of lymphatic drainage^(24)^.

In general, the history of PBM use in speech-language pathology is still incipient, unlike other professions, such as dentistry and physical therapy, whose usage guidelines are more established^(25)^. Additionally, there is a limited number of publications and robust external evidence regarding its application, specifically in the context of laryngeal tissue and musculature^(26)^. On March 17, 2021, the Brazilian Federal Council of Speech-Language Pathology (CFFa) issued Resolution no. 606 on the use of PBM devices by speech-language pathologists, regulating its use within professional practice. According to this Resolution, PBM may be used as a therapeutic device in conjunction with conventional clinical speech-language procedures^(27)^.

The incorporation of a new strategy or intervention in healthcare should be based on an understanding of the underlying principles of the strategy and the expected (or previously verified) effects in individuals with a specific health condition^(28)^. Ideally, the results of randomized clinical trials and systematic reviews with meta-analysis should be considered as the main sources of information for the clinician’s decision-making^(29)^. However, the available literature on the effects of PBM on the larynx remains scarce and presents methodological limitations^(17,30-32)^, making it challenging to base decision-making primarily on specific scientific literature.

It is essential to recall that the concept of evidence-based practice involves three main pillars: external evidence from scientific research; expert opinion; and patient preferences and values^(33,34)^. Thus, considering that sufficient external evidence on the immediate and long-term effects of PBM in voice rehabilitation and habilitation is not yet available, the current use of this device can be justified by translational strategies and analogies regarding PBM effects on other body tissues, by the clinician's perception in the therapeutic setting, and by the patient's assessment/preference regarding the perceived benefits of PBM use.

In the absence of robust external evidence, two important guidelines should be used by clinicians in their decision-making: biological and pathophysiological principles, and ethical principles^(35,36)^. In the use of PBM in therapy and vocal training, speech-language pathologists must compensate for the scarcity of scientific evidence by mastering biological and pathophysiological principles, tailoring treatments to each case of dysphonia or the needs of voice professionals, always aiming for the safe and effective use of the technology to maximize benefits and minimize risks. Essentially, the practice should be guided by bioethics, respecting patient autonomy through transparent communication, exercising non-maleficence with strict safety protocols, and promoting beneficence by fostering vocal recovery. These ethical principles ensure the clinician’s responsibility in offering PBM as part of vocal therapy and training.

In this regard, a viable alternative, in the absence of available external evidence, is the establishment of consensus among experts in the field with expertise in the use of a specific therapeutic strategy. The consensus can serve as a starting point for clinical guidance on usage criteria and as a framework for the development of future research, considering that it draws upon the clinician's biological and pathophysiological knowledge of the investigated condition, as well as the ethical principles related to the topic^(37-40)^.

Consensus methods are increasingly used as part of the development of clinical guidelines and health policies, particularly in the context of the evidence-based practice paradigm. They tend to drive changes in clinical practice by promoting engagement, consultation, and validation among peers^(39,40)^. Additionally, consensus methods are particularly important in topics where existing evidence is limited, whether it is incipient or of low methodological quality, as is the case with PBM applied to voice habilitation and rehabilitation.

The Delphi method is one of the techniques used to systematically obtain consensus on a specific topic, such as strategies applied in clinical settings but without robust scientific evidence. In health research, this method began to be used to define priority areas for investigation and funding, decision-making, and to incorporate innovative technologies and/or devices into the habilitation and rehabilitation processes of health conditions^(39)^.

Among the main advantages of the Delphi method are anonymity, the interaction of different experts, the possibility of reconsidering opinions based on controlled feedback, and the ability to achieve the primary goal, which is to resolve a problem or define a consensus on a specific topic^(38)^.

Thus, considering the scarcity of literature regarding the use of PBM in voice and the need to expand the foundation to support such practice in the area, the present research aimed to develop a consensus with expert speech-language pathologists regarding the criteria for recommending and using PBM in the context of voice habilitation and rehabilitation, through the Delphi method.

## METHODS

### Study design

This cross-sectional descriptive study aimed to develop a consensus based on the opinions of experts, using the Delphi technique. This research was submitted to the Human Research Ethics Committee at the institution of origin and was approved under evaluation report number 3.998.709. All study volunteers had access to an informed consent form and agreed to participate.

### Participants

The selection of a panel of experts is a fundamental part of the Delphi method. In summary, despite the lack of consensus on what defines an expert^(37)^, the members of the group should be committed to the project, credible, and sufficiently heterogeneous to represent a set of high-level knowledge, competencies, and skills related to the area or problem being discussed. These skills primarily depend on the experts’ knowledge^(41,42)^.

The following eligibility criteria were adopted for the selection of participants: at least 10 years of clinical experience in the field of voice, specialization in voice, a master's and/or doctoral degree in health, and the use of PBM in their clinical practice. Speech-language pathologists who did not meet these criteria were excluded^(43)^.

For recruitment, emails were sent to 11 speech-language pathologists specializing in voice and working in Brazil. The list of these professionals was provided by an institution offering both specialization and advanced training courses, classified as a teaching-learning service.

Of the 11 professionals contacted, three did not use PBM specifically in the field of voice, and one did not use the device in their clinical practice. Therefore, seven speech-language pathologists who met the eligibility criteria were selected. There is no defined number of specialists for a consensus study^(39)^. The main guideline is that the number of participants should relate to the magnitude of the problem being investigated, and there is little empirical evidence suggesting that the number of participants has a real effect on the reliability or validity of consensus processes^(44)^. In general, the development of a consensus using the Delphi technique does not require sample size calculation, as this technique relies on a series of anonymous and/or iterative questionnaires/interviews to collect opinions and information from a panel of experts. Unlike web surveys, for example, which require sample size calculation to ensure representativeness and generalization of findings, the Delphi technique does not aim for probabilistic representativeness of the population, but rather for obtaining consensus among specialists selected for their experience and knowledge in a given area^(45)^. Therefore, the most important aspect is the qualification of the participating expert^(45)^. In this sense, considering the objective of the present consensus, the absence of empirical evidence suggesting that the number of specialists affects the outcome, and the defined eligibility criteria (which aim to ensure the expert's qualification), the number reached for this research can be considered valid.

Upon confirming availability and eligibility criteria, a new email was sent to schedule a date for the first phase. Each participant was interviewed online and individually with each expert. The participants' profiles can be found in Table 1.

**Table 1:** Professional characterization of speech-language-hearing specialists who use PBM in voice

**Variable**	**N**	**%**
**SEX**		
**Females**	06	86
**Males**	1	14
**AGE RANGE**		
**31-40 years**	2	33
**41-50 years**	4	45
	1	22
**51-60 years**		
**TIME IN THE PROFESSION**		
**10-20 years**	2	28.6
**20-30 years**	5	71.4
**STATE WHERE THEY WORK AS A SPEECH-LANGUAGE-HEARING PATHOLOGIST**		
**São Paulo**	2	28.57
**Minas Gerais**	1	14.28
**Bahia**	1	14.28
**Pernambuco**	1	14.28
**Paraná**	1	14.28
**Goiás**	1	14.28
**EDUCATION LEVEL**		
**Specialization**	1	22
**Master’s degree**	4	45
**Doctoral degree**	2	33
**SPEECH-LANGUAGE-HEARING PROFESSOR**		
**Teaches postgraduate programs**	7	100
**Total**	7	100.0

To eliminate the risk of influence from the opinions of other experts, all participants remained anonymous and unaware of each other's identities throughout the process^(46)^.

### Procedures

This research used the Delphi technique because it is a systematic way to obtain consensus on a given topic from a panel of independent experts^(47)^. The Delphi technique is a structured process to list, refine, and aggregate the opinions and perceptions of a group of people (expert panel) who can contribute significantly, guiding decision-making. The Delphi technique is widely used in health studies, including consensus speech-language pathology studies^(11,48-59)^. When the technique is applied to topics with limited scientific literature based on empirical data, an initial phase of individual interviews with experts is conducted to gather the items that will be analyzed in the subsequent phases^(37,40,46,60)^.

Thus, considering the limitation in the number and quality of external evidence available for PBM application in the context of vocal therapy and training, it was decided to execute the Delphi technique in two rounds: the first round involving a semi-structured interview, allowing experts to elaborate on topics they considered important^(37,40,61)^, related to PBM as a complementary alternative in speech-language pathology intervention in the field of voice. The second round involved the judgment of each item by the participants to verify the agreement among them and the selection of consensual items.

In the therapeutic process, healthcare professionals design the intervention by considering the treatment targets, selecting the ingredients that will be used to modify those targets, and hypothesizing about the underlying mechanisms of action of the ingredients capable of promoting changes in the targets^(62)^. The specification of the ingredients includes considerations of quantity, modeling, dosimetry, progression, and variability in the execution of a specific procedure with the patient^(62)^. Thus, in both phases of the present research, the aim was to understand the specification of PBM use in voice habilitation and rehabilitation.

For the development of the items to be addressed in the interview, a literature review was conducted, in addition to the researchers' own participation based on their experience with the use of PBM. Thus, the initial version of the data collection instrument to be used in the interview included 12 questions about the use of PBM in the field of voice (Chart 1). Additionally, other questions were included that addressed the sociodemographic data and the professional profile of the participants.

**Chart 1:** PBM in-depth interview questions and voice in phase 1 of the Delphi method

Question 1 PBM is a therapeutic procedure for habilitation and rehabilitation that has been studied and reviewed in the academic field, as it holds the power of biostimulation. In general, what benefits and advances has PBM brought to Speech Therapy and to the voice area?	Determine PBM advances specifically for the Voice area.
Question 2 One of the goals of vocal therapy is to modify/manage an individual's vocal behavior and develop functional muscular adjustments adapted to the patient's vocal demands. Do you often use PBM in your clinical practice in the area of ​​voice? In which cases do you generally use PBM the most?	Highlight the use of PBM in the Voice area.
Question 3 CFFa resolution no. 541, of March 15, 2019, provides for the use of the Low-Intensity Laser device as a therapeutic device associated with conventional clinical speech-language pathology procedures, and can be used for speech-language pathology purposes. Given this prerogative, is PBM indicated for the voice area? Have you completed any training/qualification on PBM in Speech-Language Pathology with clinical parameters in the voice area? Respectively, what would the pre-practice (requirements) be for the recommendation and application of PBM in the voice area?	Denote the indication of PBM for speech therapy practice, and the prerequisites for use in the Voice area.
Question 4 Voice disorders are multidimensional and may be caused by behavioral or organic factors, or a combination of these. They represent any difficulty in vocal emission that prevents the voice from fulfilling its purpose, which is to transmit the individual's verbal and emotional message. I would like to consider three major groups of dysphonia here: behavioral dysphonia without laryngeal injury; behavioral dysphonia with laryngeal injury; and organic dysphonia. Considering these conditions, where have you used PBM most frequently?	List the cases in which the use of PBM can bring more results.
Question 5 Some studies show that speech therapy is the treatment of choice for dysphonia, especially those related to vocal behavior. The key steps for a positive treatment outcome should include guidance, awareness, and vocal training with specific techniques and exercises. Therefore, I would like to ask two questions related to the use of PBM in vocal rehabilitation: a) at what point in the rehabilitation process do you use PBM: at the beginning of the process (first patient monitoring sessions), as a complementary device to vocal training (exercises), or as an alternative for cases in which vocal training is not achieving the expected results? b) At what point in the session do you use PBM – before, during, or after performing the exercises? What are the criteria for this choice?	Highlight the moment of use of PBM in the therapeutic process.
Question 6 Regarding the use of PBM in dysphonic individuals, we have the experience of clinicians and the self-reporting of positive results from patients, but there is no scientific evidence to support the hypothesis of the effects of PBM on the voice area. What clinical guidelines do you use regarding the recommended dose for patients with inflammatory processes in the VFP? How many points per application are used in the laryngeal region? How is the effect of PBM verified: is any specific task requested before and after? Self-reporting by the client? Acoustics?	To infer which clinical parameters are related to the dose, number of points, and verification of the effect of PBM for dysphonic individuals.
Question 7 The vocal folds have distinct geometry, histology and viscoelasticity, with a complex structure that includes the lamina propria (in three layers) and the thyroarytenoid muscle, which support their biomechanical structure and phonation capacity. Regarding the wavelength, power of action and penetration of PBM, which wavelength do you use most in the larynx region?	Define the wavelength that you consider most appropriate and effective for PBM in the laryngeal region.
Question 8 The larynx is lined with mucous membrane tissue, and its walls are made of connective tissue, muscle, and cartilage. Cartilage provides support and maintains the elasticity of the larynx. According to the PBM clinical guidelines, what is the most commonly used irradiation method in the laryngeal region and what is the most commonly used anatomical site of application in the laryngeal region? Why?	Highlight the method of irradiation of PBM in the laryngeal region and the anatomical site of application.
Question 9 Regarding the use of PBM for vocal improvement/conditioning for voice professionals, studies show that the chromophores sensitized by PBM regulate components of the respiratory chain, altering their redox potential, promoting enzymatic activation and prolonging the biochemical activity of the muscle fiber. What clinical guidelines do you use regarding the recommended dose for patients undergoing vocal improvement/conditioning? I would like to consider two groups of vocal improvement/conditioning – spoken voice and improvement/conditioning – singing voice. Considering these groups, where have you used PBM most frequently? How many points per application are used in the laryngeal region? How is the effect of biomodulation verified: is any specific task requested before and after? Client self-report? Acoustics?	List the cases in which the use of PBM can bring more results. Infer the clinical parameters related to the dose, number of points, and verification of the effect of PBM for vocal improvement/conditioning.
Question 10 At what point in the qualification process do you use PBM for individuals with high vocal performance? At the beginning of the process (first patient monitoring sessions), as a complementary device to vocal training (exercises), or as an alternative for cases in which vocal training is not achieving the expected results? b) At what point in the session do you use PBM – before, during or after the exercises are performed? What are the criteria for this choice?	Outline the moment of use of PBM in the therapeutic process.
Question 11 What irradiation method and anatomical site of application of PBM do you generally use in the laryngeal region for vocal improvement/conditioning? And why?	Identify the method of irradiation of PBM in the laryngeal region and the anatomical site of application.
Question 12 The modified ILIB technique or systemic laser therapy consists of the non-invasive, continuous and indirect application of red therapeutic laser (660nm) in the region of the radial artery, continuously. This absorption by the blood leads to an increase in the metabolism and synthesis of the main physiological protein that regulates the body's oxidative system (superoxide dismutase). This enzyme inhibits the action of reactive oxygen species (ROS), leading to the protection of cells against mutations and aging, thus combating free radicals that are so harmful to health. Do you use the ILIB technique on dysphonic patients? Or for vocal conditioning/improvement?	Check the use of the ILIB technique or systemic laser therapy in the Voice area.

**Caption**: PBM = photobiomodulation; ILIB = intravascular laser irradiation of blood

The responses to the questionnaire were open-ended to obtain broader information about the perceptions regarding the topic and the scope of speech-language pathology practice in voice habilitation and rehabilitation^(63)^.

#### First round

The objective of this phase was to collect opinions, experiences, and clinical practice regarding the use of PBM in voice. The experts were invited to participate in an in-depth interview about the use of PBM in voice, based on the questions described in Chart 1. The interview lasted 40 to 60 minutes and was conducted via videoconference using the Zoom platform. They were led by the lead researcher and held individually with each participant. The interview material was recorded in audio and video for later review and transcription.

After transcribing all the interview material obtained from the seven interviewees, the content was synthesized and transformed into 55 affirmative statements. These statements were reviewed by two other researchers involved in this work, both voice specialists with training in PBM. The reviewers checked the correspondence between the affirmative statements and the original transcribed content of the interviews. This procedure aimed to reduce the risk of bias. After the review and necessary revisions, the final version of the statements is presented in Chart 2.

**Chart 2:** Statements about photobiomodulation applied to the voice area

**ITEMS**
1. – I consider photobiomodulation (PBM) indicated for use in voice.
2. – I apply PBM at the beginning of the vocal rehabilitation process.
3. – I use PBM as a complementary device to vocal training.
4. - I apply PBM as a complementary alternative only in cases where I have limitations in the results obtained using conventional vocal therapy.
5. - I believe that having training/qualification in PBM is essential for its effective use and clinical application in the voice area.
6. - I use PBM on voice professionals without dysphonia.
7. - I only use PBM in cases of vocal improvement.
8. - I use PBM in the vocal rehabilitation of dysphonic patients.
9. - I use PBM in the Voice area to improve the client/patient's muscular performance.
10. - I use PBM in the Voice area to modulate inflammation.
11. - I use PBM with voice professionals to improve muscle performance.
12. - I use PBM with voice professionals to improve muscle recovery.
13. - I use PBM with voice professionals to reduce symptoms of vocal fatigue.
14. - I use PBM in cases of patients with dysphonia due to muscle tension.
15. - I use PBM in cases of patients with laryngeal paralysis.
16. - I use PBM in cases of patients with behavioral dysphonia without laryngeal injury.
17. - I use PBM in cases of patients with behavioral dysphonia with laryngeal injury.
18. - I use PBM in cases of patients with dysphonia of organic origin.
19. - I use PBM in cases of patients with dysphonia of neurological origin.
20. - I use PBM in cases of patients with dysphonia due to sequelae of head and neck cancer.
21. - I use PBM in cases of patients with dysphonia resulting from laryngitis due to laryngopharyngeal reflux.
22. - I use PBM in the laryngeal region before performing vocal exercises.
23. - I use PBM in the laryngeal region during vocal exercises.
24. - I use PBM in the laryngeal region after performing vocal exercises.
25. - I use infrared wavelengths in the laryngeal region of patients with inflammatory processes in the vocal folds.
26. - I use point contact as a method of irradiation in the laryngeal region in patients with inflammatory processes in the vocal folds.
27. - I apply PBM to the thyroid cartilage lamina in patients with inflammatory processes in the vocal folds.
28. - I apply PBM to the anterior commissure and the thyroid cartilage lamina in patients with inflammatory processes in the vocal folds.
29. - I apply 4J (Joules) per point in the laryngeal region in patients with inflammatory processes in the vocal folds.
30. - I apply 4-6J (Joules) per point in the laryngeal region in patients with inflammatory processes in the vocal folds.
31. - I apply 6J (Joules) per point in the laryngeal region in patients with inflammatory processes in the vocal folds.
32. - I apply PBM at 3 points in the hemilarynx of patients with inflammatory processes in the vocal folds.
33. - I apply PBM at 4 points in the hemilarynx of patients with inflammatory processes in the vocal folds.
34. - I apply PBM to 5 points on the hemilarynx of patients with inflammatory processes in the vocal folds.
35. - I request a specific vocal task before and after application, to check the effect of PBM on the voice.
36. - I use patient/client self-reporting to check the effect of PBM on vocal production.
37. - I use acoustic analysis before and after applying PBM to check its effect on the voice.
38. - I use PBM as a complementary device for vocal conditioning in speaking voice professionals.
39. - I use PBM as a complementary device for vocal conditioning in singing voice professionals.
40. - I use doses lower than 4J (Joules) when my goal is to improve vocal conditioning in voice professionals.
41. - I use doses between 4-6J (Joules) when my goal is to improve vocal conditioning in voice professionals.
42. - I use doses between 6-9J (Joules) when my goal is to improve vocal conditioning in voice professionals.
43. - I use infrared wavelengths in the laryngeal region when my goal is to improve the client/patient's vocal conditioning.
44. - I use point contact as a method of irradiation in the laryngeal region when my objective is to improve the client/patient's vocal conditioning.
45. - I apply PBM at 3 points in the hemilarynx when my goal is to improve the client/patient's vocal conditioning.
46. - I apply PBM to 4 points on the hemilarynx when my goal is to improve the client/patient's vocal conditioning.
47. - I apply PBM to 5 points in the hemilarynx when my goal is to improve the client/patient's vocal conditioning.
48. - I use PBM in the laryngeal region before performing vocal exercises when my goal is vocal conditioning for voice professionals.
49. - I use PBM in the laryngeal region during vocal exercises when my goal is vocal conditioning for voice professionals.
50. - I use PBM in the laryngeal region after performing vocal exercises when my goal is vocal conditioning for voice professionals.
51. - I use point contact as a method of irradiation in the laryngeal region when my objective is vocal conditioning for voice professionals.
52. - I apply PBM to the thyroid cartilage lamina when my goal is vocal conditioning for voice professionals.
53. - I apply PBM to the anterior commissure and the keel of the thyroid cartilage in the laryngeal region when my objective is vocal conditioning for voice professionals.
54. - I use the ILIB technique on dysphonic patients.
55 - I use the ILIB technique on clients whose goal is vocal conditioning/improvement.

#### Second round

The objective of the second round was to obtain the experts' opinions regarding the dosimetric parameters and criteria for the use of PBM in voice. Participants were invited to respond to a questionnaire via Google Forms.

**Figure 1:**
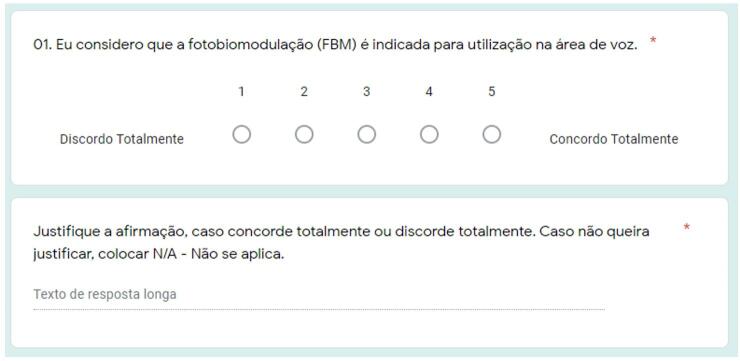
Model of sentences and Likert scale presented in the second round

The sentences obtained and formulated at the end of the first round were presented one by one to the participants, who were asked to rate their level of agreement with each statement using a five-point Likert scale, ranging from “strongly agree” (1) to “strongly disagree” (5) (Figure 1). This scale was used for its simplicity and ability to allow statistical analysis through the content validity coefficient (CVC)^(40,46)^. Additionally, participants could comment on each item to qualify their response and assist in refining the statement for future iterations^(37,47)^.

*Sentence must be answered

Caption: N/A = not applicable

All participants received the link to an informed consent form, instructions for completing this stage, and the instrument containing the 55 items of the study. Initially, participants were invited to thoroughly read the ICF and either confirm or decline their participation in the study. Upon consenting, participants were guided to the first section, which consisted of instructions for completing the instrument, followed by subsequent sections containing statements about the use of biofeedback in clinical voice practice.

After answering all questions, the specialist would activate the “submit” command, completing the data collection process. The estimated time to complete the form was approximately 15 minutes. The instrument remained open for responses for 8 weeks. Data obtained in this stage were extracted from the Google Forms platform into a Microsoft Excel spreadsheet for statistical analysis.

In this study, two rounds were chosen to obtain the consensus items^(61,64,65)^. Two rounds are recommended because participants may become fatigued from answering the same items and tend to change their responses simply to conclude their participation. Thus, at the end of the second round, the data were tabulated and subjected to statistical analysis to verify agreement and establish consensus.

### Statistical analysis

The CVC was used as the main criterion to determine consensus on each item. The CVC assesses the degree of agreement among judges in evaluating each item of the questionnaire^(66-68)^.

To calculate the CVC, the means, total scores, and error values of the Likert scale ratings (1 to 5 points for each item) were obtained for each statement in the questionnaire. For the final calculation, the CVC of each item was subtracted by the constant in the formula or error calculation. Chart 3 shows the method used to obtain the coefficient^(69,70)^.

**Chart 3:** Method used to calculate CVC

CVC formula
$1{-}_{i}\frac{\left(\frac{\sum_{i}}{n}\right)}{\max}2{-}_{i}{\left(\frac{}{n}\right)}^{n}3{-}_{iii}$

In this study, the following classification was adopted^(71)^ for interpreting CVC values: excellent (CVC ≥ 0.78), good (0.60 ≥ CVC ≤ 0.77), and poor (CVC ≥ 0.59). Given that the objective of this research is to develop a consensus regarding the use of biofeedback in voice therapy and training, only items with a CVC above 0.75 were considered an acceptable consensus^(67,72)^. Data were analyzed using SPSS software, version 22.

## RESULTS

The analyses indicate a consensus in 34 items (CVC ≥ 0.75) (Table 2).

**Table 2:** Content Validity Coefficient of items related to the use of photobiomodulation in vocal habilitation and rehabilitation

**ITEM**	**CVC**
1. I consider photobiomodulation (PBM) indicated for use in voice.	1.00*
2. I apply PBM at the beginning of the vocal rehabilitation process.	0.86*
3. I use PBM as a complementary device to vocal training.	1.00*
4. I apply PBM as a complementary alternative only in cases where I have limitations in the results obtained using conventional vocal therapy.	0.52
5. I believe that having training/qualification in PBM is essential for its effective use and clinical application in the voice area.	1.00*
6. I use PBM on voice professionals without dysphonia.	1.00*
7. I only use PBM in cases of vocal improvement.	0.71
8. I use PBM in the vocal rehabilitation of dysphonic patients.	0.86*
9. I use PBM in the Voice area to improve the client/patient's muscular performance.	0.95*
10. I use PBM in the Voice area to modulate inflammation.	0.71
11. I use PBM with voice professionals to improve muscle performance.	0.90*
12. I use PBM with voice professionals to improve muscle recovery.	0.90*
13. I use PBM with voice professionals to reduce symptoms of vocal fatigue.	1.00*
14. I use PBM in cases of patients with dysphonia due to muscle tension.	0.71
15. I use PBM in cases of patients with laryngeal paralysis.	0.62
16. I use PBM in cases of patients with behavioral dysphonia without laryngeal injury.	0.81*
17. I use PBM in cases of patients with behavioral dysphonia with laryngeal injury.	0.52
18. I use PBM in cases of patients with dysphonia of organic origin.	0.57
19. I use PBM in cases of patients with dysphonia of neurological origin.	0.48
20. I use PBM in cases of patients with dysphonia due to sequelae of head and neck cancer.	0.43
21. I use PBM in cases of patients with dysphonia resulting from laryngitis due to laryngopharyngeal reflux.	0.71
22. I use PBM in the laryngeal region before performing vocal exercises.	1.00*
23. I use PBM in the laryngeal region during vocal exercises.	0.48
24. I use PBM in the laryngeal region after performing vocal exercises.	0.81*
25. I use infrared wavelengths in the laryngeal region of patients with inflammatory processes in the vocal folds.	0.86*
26. I use point contact as a method of irradiation in the laryngeal region in patients with inflammatory processes in the vocal folds.	0.95*
27. I apply PBM to the thyroid cartilage lamina in patients with inflammatory processes in the vocal folds.	0.81*
28. I apply PBM to the anterior commissure and the thyroid cartilage lamina in patients with inflammatory processes in the vocal folds.	0.86*
29. I apply 4J (Joules) per point in the laryngeal region in patients with inflammatory processes in the vocal folds.	0.76*
30. I apply 4-6J (Joules) per point in the laryngeal region in patients with inflammatory processes in the vocal folds.	0.86*
31. I apply 6J (Joules) per point in the laryngeal region in patients with inflammatory processes in the vocal folds.	0.95*
32. I apply PBM at 3 points in the hemilarynx of patients with inflammatory processes in the vocal folds.	0.62
33. I apply PBM at 4 points in the hemilarynx of patients with inflammatory processes in the vocal folds.	0.71
34. I apply PBM to 5 points on the hemilarynx of patients with inflammatory processes in the vocal folds.	0.43
35. I request a specific vocal task before and after application, to check the effect of PBM on the voice.	1.00*
36. I use patient/client self-reporting to check the effect of PBM on vocal production.	1.00*
37. I use acoustic analysis before and after applying PBM to check its effect on the voice.	0.71
38. I use PBM as a complementary device for vocal conditioning in speaking voice professionals.	1.00*
39. I use PBM as a complementary device for vocal conditioning in singing voice professionals.	0.95*
40. I use doses lower than 4J (Joules) when my goal is to improve vocal conditioning in voice professionals.	0.33
41. I use doses between 4-6J (Joules) when my goal is to improve vocal conditioning in voice professionals.	1.00*
42. I use doses between 6-9J (Joules) when my goal is to improve vocal conditioning in voice professionals.	0.95*
43. I use infrared wavelengths in the laryngeal region when my goal is to improve the client/patient's vocal conditioning.	0.81*
44. I use point contact as a method of irradiation in the laryngeal region when my objective is to improve the client/patient's vocal conditioning.	0.90*
45. I apply PBM at 3 points in the hemilarynx when my goal is to improve the client/patient's vocal conditioning.	0.76*
46. I apply PBM to 4 points on the hemilarynx when my goal is to improve the client/patient's vocal conditioning.	0.76*
47. I apply PBM to 5 points in the hemilarynx when my goal is to improve the client/patient's vocal conditioning.	0.62
48. I use PBM in the laryngeal region before performing vocal exercises when my goal is vocal conditioning for voice professionals.	0.95*
49. I use PBM in the laryngeal region during vocal exercises when my goal is vocal conditioning for voice professionals.	0.43
50. I use PBM in the laryngeal region after performing vocal exercises when my goal is vocal conditioning for voice professionals.	0.33
51. I use point contact as a method of irradiation in the laryngeal region when my objective is vocal conditioning for voice professionals.	0.90*
52. I apply PBM to the thyroid cartilage lamina when my goal is vocal conditioning for voice professionals.	0.90*
53. I apply PBM to the anterior commissure and the keel of the thyroid cartilage in the laryngeal region when my objective is vocal conditioning for voice professionals.	0.81*
54. I use the ILIB technique on dysphonic patients.	0.62
55. I use the ILIB technique on clients whose goal is vocal conditioning/improvement.	0.71
**Total**	0.78*

* Items with CVC > 0.75

Caption: CVC = content validity coefficient

It was noted that 31 items achieved an excellent CVC (≥ 0.78), 14 items had a good CVC (0.60 ≥ CVC ≤ 0.77), and 10 items had a poor CVC (≥ 0.59). The overall CVC was considered excellent, with a value of 0.78.

## DISCUSSION

The objective of this study was to develop a consensus on clinical guidelines for the use of biofeedback in vocal habilitation and rehabilitation, aimed at supporting the professional practice of speech-language pathologists in this field and providing indicators for future research in the area. Generally, the outcome of applying consensus methods is associated with improved decision-making, development of criteria for specific practices, and provision of benchmarks in the absence of sufficient external evidence or when there is uncertainty regarding the effect and effectiveness of such practices for a specific condition^(73)^.

In this study, seven specialists participated in both rounds, with no loss of participants between rounds. The commitment of the expert panel is essential in this type of research and may reflect the level of interest in the topic^(74)^, particularly regarding the foundation of therapeutic specification of biofeedback as a complementary strategy in the vocal habilitation and rehabilitation process.

In the present study, consensus was reached on 34 items related to the use of biofeedback in the field of voice. For clarity, the consensus items were grouped into categories to facilitate the flow of information in the discussion, including general characteristics of biofeedback use in voice; selection of patients or clients for biofeedback application; therapeutic targets with biofeedback use; timing of irradiation; biofeedback dose administered; biofeedback application site; method and application points for biofeedback; wavelength used; and effect measures following biofeedback use.

### General characteristics of the use of biofeedback in voice

Six items (1, 3, 4, 5, 38, and 39) addressed issues related to the general characteristics of using biofeedback in voice. Among these, consensus was reached on five items, with the exception of item number four.

The specialists consider biofeedback to be suitable for use in the field of voice (item 1) and use it as a complementary device in vocal training (items 3, 38, and 39). Although there is no available external evidence of high quality regarding the use of biofeedback in the field of voice, specialists have been using this device in voice therapy and training. In general, speech-language pathologists have relied on studies from basic sciences and inference, drawing logical conclusions based on research results regarding its application to other structures in the head and neck region, or conclusions based on clinical reasoning from the expected effects of irradiation on body tissues.

In this regard, considering that evidence-based practice relies on the tripod of external evidence, expert opinion, and patient preferences, the use of biofeedback in the field of voice appears to be based on expert opinion and patient preferences, in the absence of available external evidence. Furthermore, it is noteworthy that CFFa Resolution no. 606 regulates the possibility of speech-language pathologists using biofeedback in the field of human voice, always as a complementary device in the process of voice therapy or training^(75)^.

Clinical expertise and patient or client preferences are essential elements for treatment personalization. In this sense, while new research is being conducted to provide a more robust evidence base (that may or may not support the use of biofeedback in different contexts of voice care), clinical experience and patient or client opinion are valuable aspects for decision-making.

The speech-language pathologists who participated in the study reached consensus on the need for specific and complementary training for using this device in vocal clinics (item 5). This finding aligns with the aforementioned Resolution, which states that speech-language pathologists may only use the biofeedback therapeutic device when they have specific and adequate training and are subject to legal responsibility in cases of incompetence, negligence, or recklessness. The existence of specific regulation is an indicator of the legitimacy of the practice and the need to ensure that professionals are adequately trained.

The specialists did not reach agreement regarding the use of biofeedback as a complementary alternative in cases where there are only limitations in the results with conventional therapy (Item 4). The lack of consensus on this item may reflect the inherent challenge of incorporating new technologies and devices into established clinical practices, especially in a field where there is a wide variety of approaches to achieve the same therapeutic goals.

The lack of consensus on the use of biofeedback as a complementary strategy may be related to the high value placed on traditional vocal therapy as the gold standard, whose effectiveness and safety are widely recognized and supported by extensive scientific literature. Additionally, caution in recommending new devices is a desirable characteristic in evidence-based practice, ensuring that new approaches are adopted based on proven results rather than novelty or trends.

On the other hand, the lack of agreement regarding the limited use of biofeedback may indicate an openness to explore its potential more broadly, not only as a device to be used in situations where conventional therapy has limitations, but also as an element to be integrated into conventional therapy.

Similarly, there was no consensus among the specialists regarding the use of ILIB (Intravascular Laser Irradiation of Blood - Systemic Vascular Photobiomodulation), either for the treatment of inflammatory processes in the vocal cords or for work on vocal conditioning/improvement (items 54 and 55).

The ILIB technique involves the intravascular red (most used) or infrared irradiation of blood. In speech-language pathology, it is performed in a non-invasive and transdermal manner, using a light beam positioned on the wrist over the radial or carotid artery. Its goal is to irradiate the bloodstream and, in doing so, stimulate the body's overall response^(76)^. There are no studies that demonstrate the primary effect of this technique for habilitation and rehabilitation in voice^(76)^. Therefore, the lack of consensus may be related to the absence of robust scientific evidence regarding its use for the specific purpose of vocal enhancement or rehabilitation.

In summary, the consensus result in this subsection on the general characteristics of using biofeedback in voice reinforces the need for a more nuanced approach to adopting complementary devices in conventional vocal therapy. Both respect for traditional methodologies and openness to innovation should be considered. Careful consideration of how and when to integrate new approaches could lead to a more holistic and personalized treatment paradigm, where biofeedback is not viewed as a last resort, but as a valuable part of a diversified therapeutic repertoire.

The consensus on the potential use of biofeedback in the field of voice as a complementary device to conventional therapy highlights the multimodal nature of voice therapy and training, where different elements can be utilized to achieve the best possible outcome for the patient or client.

### Selection of patients or clients for the application of biofeedback

Ten items (6, 8, 14, 15, 16-21) addressed the criteria for selecting potential patients or clients for the use of biofeedback in voice, with three items reaching consensus (items 6, 8, and 16).

The specialists agreed to use this device in vocal training for voice professionals without dysphonia and in the rehabilitation process of dysphonic patients. The justification for such use may be associated with its primary effects. Studies in the field of physical therapy with rats and humans, as well as consensus in orofacial motricity, confirm that radiation in the visible and near-infrared spectrum acts as a biomodulating agent, capable of promoting anti-inflammatory and analgesic effects through the induction of cellular and systemic responses. Taking this into account, the hypothesis is formulated, which may be the basis for the reasoning that led to the consensus on these items, regarding the potential improvement of an individual's vocal performance, particularly when subjected to high vocal demands^(22,26,77-79)^.

More specifically, regarding the use of this device in dysphonic patients, there was agreement on its use in patients with behavioral dysphonia without laryngeal lesions. Behavioral dysphonia is multifactorial; however, its primary etiology is improper vocal use, combined with exposure to risk factors for voice disorders^(80)^. It is characterized by increased muscular activity with tension and effort in intrinsic laryngeal muscles, and at times, recruitment of intrinsic laryngeal muscles during phonation. Possibly, this agreement among the specialists is associated with the increase in muscle ATP and, consequently, the reduction of muscle fatigue attributed to biofeedback^(78,81)^. A study investigating the effect of biofeedback on vocal fatigue attenuation after an overload task concluded that the use of biofeedback is potentially effective in reducing fatigue and may have significant clinical relevance for populations with voice disorders or occupations involving high vocal demand^(17)^.

No consensus was reached among the specialists regarding the use of biofeedback with the objectives of modulating inflammation in cases of muscle tension dysphonia, behavioral dysphonia with laryngeal lesions, laryngeal paralysis, or in cases of laryngitis due to laryngopharyngeal reflux, organic and neurological dysphonia, and individuals with sequelae from head and neck cancer. In parallel to the absence of robust scientific evidence supporting the application of biofeedback in these cases, authors point out that there may be restrictions on the use of biofeedback in pre-cancerous and/or cancerous conditions, as it could induce the proliferation of cancer cells^(82-87)^.

The lack of consensus regarding the use of biofeedback in a variety of other vocal conditions, including muscle tension dysphonia with laryngeal lesions, laryngeal paralysis, laryngitis due to reflux, organic and neurological dysphonia, and individuals with sequelae from head and neck cancer, signals caution among the specialists. This may be attributed to the lack of robust evidence and concerns about safety, particularly regarding the potential for cancer cell proliferation. The specialists' conservative stance could reflect an ethical responsibility to avoid exposing patients to treatments whose benefits and safety are not yet clearly established in the scientific literature. Additionally, the lack of consensus on specific vocal conditions may indicate that the specialists do not yet have well-defined hypotheses and criteria on how such conditions specifically respond to biofeedback.

The lack of consensus among specialists about using biofeedback solely for vocal enhancement raises pertinent questions regarding the applicability and limits of interventions involving this device. From a clinical perspective, the specialists' caution can be interpreted as a reflection of the still-emerging nature of biofeedback within the field of vocal training. The specialists may be considering that biofeedback, in essence, is a device with specific therapeutic indications, rather than one focused directly on vocal enhancement. From a pragmatic standpoint, incorporating a new device into established practices requires training, costs, and a learning curve that may not be justified for vocal enhancement, especially if less invasive and more traditional methods with proven results are available.

Additionally, the use of the word “only” may have connoted a limitation that does not align well with the specialists' broader training approach, which may favor a more integrative perspective, viewing biofeedback as part of a larger set of strategies for vocal enhancement.

The lack of consensus may also reflect hesitation in approving biofeedback for vocal enhancement due to the absence of robust evidence in this specific context, as well as the scarcity of such evidence across the entire field of voice. Therefore, the discussion surrounding the use of biofeedback solely for vocal enhancement remains an area of active debate. Ultimately, the decision to use biofeedback for vocal enhancement should be made based on a careful consideration of the clinical, ethical, and practical implications, always with a keen eye on the evolution of research and professional guidelines.

This lack of consensus in the field of vocal enhancement may indicate that specialists have a greater understanding or preference for using the device with a primarily therapeutic focus, rather than as a tool specifically aimed at vocal improvement. This distinction is crucial, as it implies a reflection on the indications and limitations of biofeedback.

Therefore, the discussion regarding the applicability of biofeedback for vocal enhancement remains open and active, highlighting the need for a careful evaluation of the clinical, ethical, and practical implications. The decision to employ biofeedback for this purpose should be grounded in a thorough analysis of the existing literature, as well as the ongoing evolution of research and professional guidelines, ensuring that the practices adopted are aligned with the best available evidence and the principles of patient care.

On the other hand, a study conducted with 148 Brazilian speech-language pathologists working in voice revealed that they use this device in cases of behavioral dysphonia, regardless of the presence of vocal fold lesions, as well as in individuals with professional voice use in neurological dysphonia. This indicates that the specialists selected for the present research adopted a more conservative stance regarding the patients/clients eligible for the use of biofeedback.

### Therapeutic goals with PBM use

Of the six points addressing therapeutic targets in the use of biofeedback (items 7, 9, 10, 11-13), four reached consensus (items 9, 11-13). These pertain to the improvement of performance and muscle recovery, as well as the reduction of vocal fatigue symptoms in the client/patient, including voice professionals. There was no consensus regarding the use of this device for inflammation modulation.

The findings in question address a central and current topic in the field of speech-language pathology and voice therapy: the use of PBM as a device to improve vocal performance and assist in muscle recovery, as well as in reducing vocal fatigue symptoms. These aspects are of particular interest to voice professionals, whose careers are intrinsically dependent on healthy and functional voice production.

Improving vocal performance and muscle recovery are key goals in vocal rehabilitation and the training of voice professionals. PBM, based on the studies that support it, is presented as a promising intervention due to its potential biomodulatory effects. In the field of sports science, evidence suggests that the use of PBM is related to increased muscle performance and reduced fatigue due to its mechanism of action^(14-17,31,88-91)^. As results from these studies show^(14-17,92-94)^, PBM accelerates oxidative chain reactions at the mitochondrial level, enhances microcirculation, improves lymphatic drainage, and promotes the proliferation and mobility of satellite cells, speeding up collagen synthesis and reducing the inflammatory response, as well as promoting effective tissue healing^(14-17,31,88-91)^. The only intervention study in the field of voice that investigated the effect of PBM on attenuating laryngeal fatigue after vocal overload found that its use significantly normalized a combination of measures such as phonatory threshold pressure, inability to produce a smooth voice, and relative fundamental frequency, after a significant increase caused by the vocal overload task^(17)^. In general, Brazilian speech-language pathologists have used PBM for therapeutic goals related to vocal function, musculoskeletal function, and somatosensory function^(10)^.

However, the debate regarding the indication of PBM in inflammation modulation has not reached a consensus, highlighting the complexity and the need for a deeper analysis of the available evidence and clinical practice. Inflammation is a complex and multifaceted process that can be part of a protective response and tissue repair, but when dysregulated, it can also lead to damage and dysfunction. The uncertainty about using PBM for inflammation modulation may reflect the variability of results presented by studies, possible heterogeneity in research methodologies, or a lack of high-quality studies providing conclusive evidence. The disagreement among experts may indicate that clinical experience and patient expectations are not aligned with research data or that the interpretation of these data varies significantly among professionals.

Additionally, the decision to apply or not apply PBM in the context of vocal inflammation may be influenced by several factors, including the type of dysphonia, the etiology of the inflammation, the stage of the laryngeal injury, and the specific conditions of the patient. The complexity of these factors may hinder the attainment of a consensus on the generalized applicability of PBM for all cases of inflammatory processes in the vocal cords.

### Timing of irradiation

Five items reached consensus regarding the timing of irradiation (items 2, 22, 24, 48, and 51). The consensus refers to the application at the beginning of the vocal rehabilitation process, and before and after performing vocal exercises in different situations, specifically in the laryngeal region, with various clients/patients, including voice professionals. There was no consensus regarding the use of PBM during and after vocal conditioning exercises (items 23, 49, and 50).

The consensus reached by the specialists regarding the timing of irradiation (at the beginning of vocal rehabilitation, as well as before and after performing vocal exercises) may reflect a trend to recognize its potential benefits in preparing and recovering the muscles involved in voice production. The decision to apply PBM at the beginning of therapy may be grounded in the hypothesis that preparing the laryngeal tissue could increase the effectiveness of subsequent vocal exercises. By improving local oxygenation and circulation, PBM may enhance the muscular response, promoting a more receptive state for training and, consequently, optimizing rehabilitation outcomes. Additionally, by applying PBM before and after vocal exercises in different situations, the specialists may be considering the device's ability to reduce muscle fatigue symptoms and promote recovery. A survey conducted among Brazilian speech-language pathologists^(10)^ showed that they tend to use PBM before performing vocal exercises, which aligns with the findings of this consensus.

The lack of consensus regarding the application of PBM during and after vocal exercises. However, reveals an area of uncertainty and debate. The hesitation to recommend PBM concurrently with exercises may stem from concerns about the possibility of overstimulating the laryngeal tissues, which could theoretically lead to adverse effects or reduce the effectiveness of the exercises. On the other hand, the movement in the laryngeal framework caused by most vocal exercises could reduce the accuracy of the irradiation point chosen by the clinician. The absence of agreement may also be attributed to the limited research examining the immediate effects of PBM on laryngeal tissues during activity.

Regarding the application of PBM after vocal exercises, it may be an area of divergence due to the complexity of the physiological responses involved in muscle recovery. A study^(17)^ demonstrated the metabolic and photochemical effects of PBM on the vocal folds, promoting adequate phonation with improvements in acoustic, aerodynamic, and perceptual-auditory measures, in addition to aiding muscle recovery after exertion, favoring energy supply for muscle balance after vocal exercises. However, the specialists may be seeking more evidence to demonstrate the effectiveness of PBM at this stage, ensuring that its use is not only safe but also effectively contributes to the improvement of vocal function.

This scenario suggests the need for more research specifically addressing the timing of PBM application in relation to vocal exercises, as well as studies that explain the underlying mechanisms of its effects on the laryngeal musculature. In the meantime, clinical practice should be guided by a combination of scientific evidence, clinical experience, and the individual needs of patients, always maintaining a conservative approach concerning patient safety and treatment efficacy.

### PBM dose

There was consensus among the specialists for doses starting at 4J per irradiation point (Items 29, 30, 31, 41, 42). The specialists reached a consensus on a dose of 6J per irradiation point for treating inflammatory processes and 9J per irradiation point for vocal conditioning. There was no consensus on doses lower than 4J per irradiation point (Item 40).

The findings regarding the consensus among specialists on PBM doses as part of vocal therapy associated with inflammatory processes and vocal conditioning indicate an effort to standardize the application of this device in voice. The agreement on minimum doses of 4J per irradiation point and the specification of higher doses for different therapeutic objectives reflect guidance based both on clinical experience and emerging evidence from other areas of healthcare.

The decision to adopt a dose of 4J or 6J per irradiation point for inflammatory processes and 9J for vocal conditioning suggests that the specialists recognize the need for sufficiently potent doses to achieve the desired therapeutic effects. The 6J dose may have been chosen as a balanced point aimed at optimizing the anti-inflammatory and analgesic effects of PBM, based on the evidence that lower doses can stimulate mitochondrial homeostasis and accelerate tissue healing^(24,95)^.

On the other hand, the 9J dose per irradiation point for vocal conditioning may be related to the effects of PBM on bioenergetic pathways and the enzymatic modulation of muscle fibers. The effectiveness of PBM in this context is intrinsically linked to its ability to penetrate muscle layers and induce beneficial biochemical responses. Higher doses may be necessary to reach deeper layers of muscle tissue, thereby promoting biochemical stimuli that enhance muscle performance and increase resistance to fatigue – key aspects for vocal conditioning, especially among professionals who rely heavily on their voice^(96,97)^. In this sense, it is essential to consider the relationship between the wavelength used in PBM and its penetration power in the tissue, as both factors determine the optimal dosage needed to achieve the desired therapeutic effects. A precise adjustment of the wavelength can optimize the light's penetration into muscle tissue, suggesting that a more detailed approach in selecting the dosage may enhance the benefits of PBM for vocal conditioning^(96)^. This reflection on the interdependence between wavelength, penetration power, and dosage is crucial to support our hypothesis with a biological basis and the best available scientific knowledge.

In the present study, the specialists did not mention the use of doses lower than 4J in the vocal therapy process for dysphonic patients during the first round of interviews, so this dosage was not included as a judgment item in the consensus analysis. This may reflect an understanding that subtherapeutic doses may not be sufficient to induce the necessary biological effects in the laryngeal tissues, either in therapy or vocal training. This does not exclude the possibility that lower doses could have some utility in specific contexts; however, the specialists seem to lean toward an efficacy threshold that ensures a perceptible clinical response.

The interpretation of these findings requires an integrated perspective that considers the underlying biological mechanisms of inflammation and vocal physiology, as well as the tissue response to irradiation. Standardizing PBM doses represents an important step in the development of more effective and safe treatment protocols for vocal rehabilitation. However, it is crucial that these recommendations are continuously re-evaluated considering new scientific evidence and reflective clinical practice.

The present consensus, although based on clinical experience and the transfer of findings from related fields, highlights the need for future clinical trials to confirm the efficacy and safety of the stipulated doses. Such studies must be rigorous, controlled, and replicable, so that the scientific community can fully trust the dosage recommendations and clinicians can apply PBM with the highest possible precision in their treatments.

The apparent discrepancy in the research results, where there was no consensus among specialists regarding the use of PBM for modulating inflammation in the vocal folds, in contrast to a broader consensus on specific doses starting from 4J per irradiation point and the use of infrared wavelength for patients with inflammatory processes, may initially seem paradoxical. However, a more detailed analysis of these findings may reveal an underlying logic that clarifies this apparent contradiction.

First, the lack of consensus regarding the use of PBM for modulating inflammation may reflect a general caution among specialists about the universal applicability of PBM for all cases of inflammation in the vocal folds. This caution may be attributed to the complexity of inflammatory processes, which can vary significantly in severity, etiology, and response to treatment. Additionally, the heterogeneity in patients' clinical conditions and the specific characteristics of inflammation may lead specialists to adopt a more individualized approach, considering PBM as one treatment option among several others, depending on the specific clinical context of each patient.

On the other hand, the broader consensus regarding specific doses and infrared wavelength suggests that once the decision to use PBM is made, specialists may prefer certain irradiation doses. This indicates that, for cases where PBM is considered appropriate, there is strong agreement on how it should be applied to maximize efficacy and minimize risks. Therefore, the apparent discrepancy in the results reflects a pragmatic approach, based on hypotheses about the effects of the doses used by the specialists.

### Location of application

Regarding the location of PBM application in the laryngeal region, the specialists reached consensus on applying PBM to the thyroid cartilage lamina, the anterior commissure, and the keel of the thyroid cartilage, both for inflammatory processes in the vocal folds and for conditioning situations in voice professionals (Items 27, 28, 52, and 53).

The choice of these specific areas for PBM application is based on the anatomy of the vocal folds and their relevance to vocal physiology^(98)^. The thyroid cartilage lamina and the anterior commissure are crucial anatomical points, providing more direct access to the vocal folds^(99,100)^ and, consequently, to the tissues that may benefit from the effects of PBM. Anatomically, the vocal folds extend horizontally in the larynx, with the body running parallel to the entire length of the thyroid laminae, having an anterior fixation on the inner surface of the thyroid cartilage (forming the anterior commissure), and a posterior fixation on the vocal and muscular processes of the arytenoid cartilages^(101,102)^. The vocal folds are histologically composed (medio-laterally) of five layers: stratified epithelium, superficial, intermediate, and deep layers of the lamina propria, and the thyroarytenoid muscle^(103)^.

It is important to note that for PBM to be effective, the irradiation must reach the entire extent of the target area. This is crucial because the vocal folds are made up of different layers that work together to produce voice^(102)^. The irradiation needs to penetrate these layers adequately to promote a biomodulatory effect that can positively influence cellular metabolism, reduce inflammation, and aid in tissue recovery^(81,104)^.

The thyroarytenoid muscle, which constitutes the body of the vocal folds, is particularly relevant in the discussion of PBM application^(99)^. Hypothetically, this is the first intrinsic muscle to receive light irradiation due to its lateral position. The efficacy of PBM in improving the function of this muscle may have significant implications for the vocal production process. Stimulating this muscle can help reduce muscle tension^(98,99,105)^, which is essential for the recovery of inflammatory processes and for vocal conditioning, especially in voice professionals who rely on the endurance and flexibility of this muscle for optimal performance.

The agreement among the specialists on the PBM application sites also demonstrates the recognition of the importance of following well-established irradiation parameters. Factors such as the target, application method, wavelength, dose, power, power density, emission type, energy density, total energy, and time are all critical to ensuring that the treatment is not only effective but also safe for the patient^(89)^.

In summary, the consensus among the specialists regarding the location of PBM application in the laryngeal region is based on a deep understanding of the anatomy and physiology of the vocal folds, as well as the need to target therapy to maximize benefits while minimizing risks. As research progresses, it is likely that further refinements in PBM treatment parameters will be established, contributing to an even more effective and informed clinical practice.

### Mode and points of PBM application

Regarding the PBM application techniques, the specialists reached a consensus on the use of point contact as the method of irradiation in patients with inflammatory processes in the vocal folds and when the therapeutic goal is to improve vocal conditioning (items 26, 44, and 51). The experts agreed on applying PBM at three or four points on the hemilarynges when the goal is to enhance the vocal conditioning of the client/patient (items 45, 46). However, there was no consensus regarding the application of three to five points on the hemilarynges of patients with inflammatory processes in the vocal folds, nor on applying PBM to five points on the hemilarynges of clients whose objective is to improve vocal conditioning (items 32-34 and 47).

The consensus around the use of the point contact technique as an irradiation method highlights the importance of precision in the application of PBM, as well as the need for a targeted approach that considers the anatomical and functional peculiarities of the laryngeal region^(100,101,106,107)^. Point contact was agreed upon by the specialists due to its ability to minimize dispersion and energy loss, ensuring that the irradiation dose is delivered more efficiently to the target tissue. This technique is considered the most suitable for reaching the laryngeal structures, particularly the vocal folds and the intrinsic musculature, which are crucial for voice production^(17,81)^. Related literature indicates that point contact with a 90° angle of incidence perpendicular to the target is the most appropriate method, as it reduces the possibility of energy loss through reflection^(81)^. It is likely that voice specialists follow this recommendation based on these studies.

The decision to apply PBM at three or four specific points in the hemilarynx to improve vocal conditioning may reflect a strategic approach to cover the most relevant areas for vocal function, without overloading the region with unnecessary irradiation points.

The lack of consensus on the application at three to five points for inflammatory processes and five points for vocal conditioning may be attributed to several factors. On one hand, there may be a consideration that a higher number of irradiation points may not provide significant additional benefits, and that a more focused approach may be sufficient to achieve the desired effects. On the other hand, the debate may be related to concerns about overexposure and the potential for adverse effects, especially in tissues already compromised by inflammatory processes.

It is important to note that precise PBM application is crucial to ensure the desired therapeutic effects. Imprecise positioning of the irradiation points can not only reduce the effectiveness of the treatment but also increase the risk of undesirable effects^(92)^. The laryngeal region is complex^(100,107)^, and precision in the application of PBM is essential to properly stimulate the targeted structures without affecting the surrounding tissues.

In addition, the application parameters, including the number of points and the application technique, must be carefully chosen to reflect the specific therapeutic objectives, whether to reduce inflammation or to improve vocal conditioning. The penetration ability and the immediate beneficial effects of PBM are critical aspects that can enhance the therapeutic process and, therefore, should be considered when designing the treatment plan^(108)^.

In summary, the consensus on the application technique of PBM in the laryngeal region indicates a careful and methodical approach, considering both patient characteristics and the anatomical and functional specifics of the vocal folds. The discussion surrounding the ideal number of irradiation points reflects an area of ongoing investigation, requiring more research to optimize treatment protocols. Continuous collaboration between research and clinical practice is essential to refine PBM techniques, ensure the best possible outcomes for patients, and advance evidence-based practice in the field of speech-language pathology and vocal therapy.

### Wavelength

The specialists reached a consensus regarding the use of infrared wavelength in the laryngeal region, both in patients with inflammatory processes in the vocal folds and in individuals whose goal is to improve vocal conditioning (Items 25 and 43).

The choice of infrared wavelength for applications in the laryngeal region, both for inflammatory processes in the vocal folds and for improving vocal conditioning, is based on the deeper penetration ability of this light spectrum^(93,94,109)^. Infrared light can penetrate beyond the superficial barriers of the skin, reaching deeper tissues such as the intrinsic muscles of the larynx and the layers of the vocal folds^(109)^.

In cases of inflammatory processes, the use of infrared aims to promote an anti-inflammatory and analgesic effect in depth, relieving symptoms and accelerating the healing process. Given the ability of infrared light to reach the dermis and underlying tissues^(24,110,111)^, specialists may have recognized its potential as an adjuvant to modulate inflammatory processes in the vocal folds.

For vocal conditioning, especially in voice professionals, the choice of infrared light can be attributed to its potential to stimulate cellular metabolism and promote faster recovery of tissues subjected to constant and intensive use^(109)^. The energy provided by infrared light can help optimize muscle performance, increasing the resistance and capacity of the vocal folds to withstand recurrent vocal stress.

The application of PBM with infrared light in the laryngeal region therefore reflects an understanding of the complex interaction between light and biological tissues, as well as an appreciation of the mechanisms by which PBM may offer therapeutic benefits. The consensus among experts highlights the importance of an approach within the available scientific knowledge that recognizes the 'optical window' of human tissue as a crucial determinant for the efficacy of the intervention^(93,94,109)^.

However, it is important to emphasize that the effect of PBM depends not only on the wavelength, but also on a series of carefully selected optical parameters, such as fluence, power density and pulse structure^(112,113)^. It is the combination of these parameters that will determine the optimal dose-response and avoid subtherapeutic or potentially harmful effects.

In summary, the consensus on the use of infrared wavelengths in speech-language pathology for laryngeal treatment demonstrates the integration of advanced knowledge of light-tissue interaction and an innovative clinical application that seeks to optimize therapeutic results for both vocal pathologies and the conditioning of healthy individuals. This consensus also serves as an impetus for additional research that may confirm or refute existing hypotheses, further solidifying the scientific basis of PBM in voice.

### Effect measures after PBM use

To verify the effect of PBM on the voice, experts requested a specific vocal task before and after application, as well as the patient/client's report (items 35 and 36). There was no agreement regarding the use of acoustic analysis for the same purpose (item 37).

Understanding interventions using PBM in voice therapy involves the use of assessment methods that can provide concrete data on the therapeutic effects of this device. The consensus among experts regarding the use of specific vocal tasks and the subjective report of the patient/client before and after the application of PBM to the voice reflects the recognition of the need for a functional and experiential assessment to measure therapeutic results.

The request for a specific vocal task before and after (which is also widely used under the term “therapeutic trial”) the application of PBM allows a direct comparison of vocal function^(7,114)^. Such tasks may include the production of sustained sounds, vocal scales, or spoken or sung phrases, which aim to assess vocal quality, vocal range, voice projection capacity, and other functional parameters. This method of assessment is crucial to understanding how PBM can affect the biomechanics and aerodynamics of voice production and provide an immediate and noticeable improvement in vocal function^(115)^.

Furthermore, patient/client reporting is equally important as it provides a subjective dimension of the impact of PBM. The individual’s perception of changes in their voice, comfort when speaking or singing, and the presence of symptoms such as pain or vocal fatigue are fundamental aspects that cannot be captured by objective measures alone^(114,116-118)^. These subjective reports are essential for a holistic understanding of the effects of PBM on the patient.

However, the lack of agreement on the use of acoustic analysis may be related to several issues, such as the accessibility and complexity of the necessary equipment, the variability in measurements due to individual differences or environmental conditions, or even the lack of consensus on which acoustic parameters are most relevant for evaluating the effectiveness of PBM^(115,119)^. Acoustic voice analysis provides objective data on the sound characteristics of the voice and is a valuable tool in voice research and clinical practice, as it can detect subtle changes in the voice that may not be perceptible to the human ear or the patient^(115)^.

A scoping review that analyzed the most used vocal assessment measures to evaluate the effect of training on healthy voices identified acoustic analysis as the first option and self-assessment as the third most used by speech-language pathologists specializing in voice^(120)^. Thus, future studies need to define the main clinical outcomes to be considered to evaluate the effectiveness of PBM in the voice area.

## LIMITATIONS AND DIRECTIONS FOR FUTURE RESEARCH

This consensus study brings important contributions to speech-language pathology, especially in the use of PBM in vocal therapy and training, marking an advance in the standardization of clinical procedures and reinforcing evidence-based practice. The integration of expert opinion and patient preferences, even in the absence of robust evidence, aligns with the tripartite model of evidence-based practice, which values ​​external evidence, clinical experience, and patient needs. The increasing complexity of clinical demands requires that practice and research evolve together, suggesting the need for future studies focused on expanding the body of external evidence through randomized controlled trials^(37,45,121)^.

The lack of robust scientific evidence on PBM in voice therapy implies a greater reliance on the specialist knowledge of speech-language pathologists to tailor treatment to individual patient needs. Implementation of PBM should be guided by an understanding of the biological mechanisms of the voice and bioethical principles, emphasizing transparency about potential benefits and risks. This study establishes important guidelines but recognizes the continued need for evidence-based research to refine the application of PBM in voice practice.

The descriptions generated from this study can serve as support for clinical speech-language pathologists who work with vocal habilitation and/or rehabilitation to obtain a standard for how this device has been used by specialists in the field. It also serves speech-language pathologist researchers, so that they can transform the items of this consensus into research hypotheses, so that their effectiveness or ineffectiveness can be tested and proven.

In implementing PBM for voice therapy and training, clinicians must navigate a field with limited scientific evidence, emphasizing understanding of the biological and pathophysiological mechanisms of voice and strictly adhering to bioethical principles. Transparency with patients regarding potential benefits and risks is essential, as is ensuring that light dosages are safe and effective. Beneficence is a key goal, seeking to promote vocal health and recovery from injury with informed use of PBM, while justice is manifested in equitable access to these therapies. Commitment to ethics and scientific knowledge allows clinicians to practice responsibly in vocal rehabilitation with PBM, despite the evident limitations of current research.

In summary, the guidelines established in this study are a milestone in the practice of speech-language pathology in the field of voice; however, the field must continue to seek a deeper, evidence-based understanding of the application of PBM, adapting as new information becomes available.

## CONCLUSION

There was consensus regarding the indication of PBM as a complementary device to conventional vocal therapy and training. The experts agreed that PBM can be used in voice professionals without dysphonia and in dysphonic patients, specifically in cases of behavioral dysphonia without laryngeal injury. The therapeutic targets associated with the use of PBM involved improved muscle performance and recovery and decreased symptoms of vocal fatigue in the client/patient, including voice professionals. The experts consider that PBM should be applied at the beginning of the rehabilitation process, before and after the performance of vocal exercises. The experts reached a consensus on the dose of 4J and 6J for each irradiation point for the treatment of inflammatory processes and 9J per irradiation point for vocal conditioning. There was consensus regarding the application of PBM in the lamina of the thyroid cartilage, in the anterior commissure and in the keel of the thyroid cartilage, both for situations of inflammatory process in the vocal folds and for situations of vocal conditioning of voice professionals. The use of point contact was a consensus among the experts, with application in three and/or four points, in the hemilarynx, when the objective is to improve the vocal conditioning of the client/patient. The experts reached consensus regarding the use of infrared wavelength in the laryngeal region, both in patients with inflammatory processes in the vocal folds, and in individuals whose objective is to improve vocal conditioning. The experts agreed on the use of a vocal task before and after administration of PBM, as well as the patient/client's report to evaluate the effect of PBM.
