# Supplementary material for: Photobiomodulation on vocal training and rehabilitation: Delphi consensus based on experts
Source: Codas. 2025 Feb 5;37(2):e20230356. doi: 10.1590/2317-1782/e20230356en (PMC11906107; doi:10.1590/2317-1782/e20230356en)
Supplement: Translation

# Translation: Fotobiomodulação na habilitação e reabilitação vocal: consenso Delphi baseado em especialistas

**DOI:** 10.1590/2317-1782/e20230356pt

**Authors:** Emerson Soares Pontes, Thays Garcia Vaiano, Roberto Sávio de Assunção Bastos, João Marcos da Trindade Duarte, Émile Rocha Santana, Leonardo Wanderley Lopes

**Affiliations:** 1 Universidade Federal da Paraíba – UFPB - João Pessoa (PB), Brasil.; 2 Centro de Estudos da Voz – CEV - São Paulo (SP), Brasil.; 3 Hospital Pronto Socorro Municipal Mário Pinotto - Belém (PA), Brasil.; 4 Universidade do Estado da Bahia – UNEB - Salvador (BA), Brasil.

**Keywords:** Voz, Qualidade da voz, Treinamento da Voz, Distúrbios da Voz, Terapia Com Luz de Baixa Intensidade, Técnica Delfos

## Abstract

**Objetivo:**

desenvolver um consenso de fonoaudiólogos especialistas em voz, acerca dos critérios de recomendação e uso da fotobiomodulação (PBM), no contexto da terapia e treinamento vocal.

**Método:**

Participaram sete fonoaudiólogos, especialistas em voz, com experiência no uso da PBM em terapia e treinamento vocal. Utilizou-se a técnica Delphi para obter o consenso dos especialistas, acessados de maneira independente em duas fases de coleta. Na Fase 1, os especialistas foram contatados individualmente e participaram de uma entrevista com 12 questões, para obtenção das opiniões quanto à utilização da PBM no contexto investigado. As respostas dos especialistas foram utilizadas para construção de um questionário com 55 itens apresentados como afirmações. Os especialistas deveriam analisar cada item e demonstrar seu nível de concordância em uma escala Likert de cinco pontos. Utilizou-se o coeficiente de validade de conteúdo (CVC) para investigar o grau de concordância entre os juízes e selecionar os itens finais do consenso.

**Resultados:**

Houve consenso entre os especialistas em 34 itens investigados nesta pesquisa, com CVC ≥ 0,75. Foi possível observar que 31 itens obtiveram excelente CVC (≥ 0,78), 14 itens com bom CVC (0,60 ≥ IVC ≤ 0,77) e 10 itens com CVC ruim (≤ 0,59). O CVC total foi considerado excelente, com valor igual a 0,78.

**Conclusão:**

Houve consenso entre os especialistas acerca do uso da PBM na habilitação e reabilitação vocal. tem potencial para melhorar os critérios de prescrição e uso desse dispositivo pelos fonoaudiólogos. Os achados podem ser úteis para melhorar os critérios de prescrição e o uso desse dispositivo pelos fonoaudiólogos, além de subsidiarem o desenvolvimento de futuras pesquisas e recomendações clínicas na área.

## INTRODUÇÃO

De maneira geral, a intervenção fonoaudiológica no campo da voz pode incluir a reabilitação das disfonias ou o treinamento para o aperfeiçoamento e condicionamento da voz falada e cantada. No contexto da reabilitação, a terapia vocal é indicada como tratamento de eleição, principalmente nos casos em que há claro envolvimento do comportamento vocal como fator etiológico, agravante ou mantenedor da disfonia^(1-8)^. A terapia vocal, realizada por fonoaudiólogos, busca modificar ajustes respiratórios, fonatórios e ressonantais, além de promover mudanças nos comportamentos vocais ineficientes aprendidos no decorrer da vida. De maneira geral, a terapia vocal envolve orientação sobre saúde vocal, conscientização acerca da psicodinâmica vocal e treinamento vocal com utilização de exercícios e dispositivos para modificação dos ajustes motores envolvidos na produção da voz^(1-9)^. Em síntese, todas as estratégias utilizadas na terapia vocal têm por objetivo desenvolver a melhor voz possível para as demandas do paciente/cliente.

Nesse contexto, os fonoaudiólogos podem dispor de dispositivos para mediar a sua ação clínica para modificação dos alvos terapêuticos e otimizar o processo de reabilitação ou habilitação^(10)^. Estes podem ser classificados em volitivos, quando demandam uma ação ou participação ativa do paciente, e não volitivos, quando não demandam uma ação ou comportamento específico do paciente^(11)^. A fotobiomodulação (FBM) é classificada como um dispositivo não volitivo, uma vez que a sua utilização, em geral, não envolve nenhuma tarefa fonatória específica solicitada de maneira concomitante à sua aplicação.

A FBM é um procedimento não invasivo, que promove bioestimulação da área irradiada devido à interação da luz visível vermelha e infravermelha absorvida pelos cromóforos endógenos. A luz absorvida desencadeia reações biológicas em nível mitocondrial, promovendo eventos fotofísicos e fotoquímicos no tecido biológico^(12)^.

De modo geral, a FBM tem sido utilizada com o objetivo de promover o reparo tecidual, modular processos inflamatórios ou produzir analgesia na região irradiada^(13,14)^. Na área de voz, a FBM é uma estratégia complementar na reabilitação de pacientes disfônicos ou no trabalho de treinamento/condicionamento de vozes profissionais^(15-17)^.

No âmbito do condicionamento vocal, seja na voz falada ou cantada, um dos principais objetivos do treinamento vocal é melhorar a performance do indivíduo em função da demanda requerida, o que envolve o aperfeiçoamento do desempenho muscular e diminuição das manifestações relacionadas à fadiga vocal^(18)^. O uso da voz em condições adversas e com recrutamento de ajustes disfuncionais no sistema de produção vocal, associado a questões como uso prolongado e em intensidade elevada, podem gerar fadiga vocal. Essa condição é, geralmente, caracterizada pela diminuição da eficiência fonatória, associada ao hiperfuncionamento vocal e à autorreferência do aumento da sensação de esforço, como resposta à determinada demanda vocal^(19-21)^.

A aplicação do FBM em indivíduos com distúrbios de voz está associada ao raciocínio clínico de que a maioria das lesões fonotraumáticas envolvem alterações edematosas e processos inflamatórios nas pregas vocais. A irradiação por FBM pode agir como modulador do processo inflamatório influenciando fatores tais como: aumento da microcirculação local^(17)^; aumento da síntese de ATP^(22)^; promoção de angiogênese e vasodilatação^(16)^; inibição de mediadores inflamatórios, como a prostaglandina e cicloxigenase; redução de espécies reativas de oxigênio e citocinas pró-inflamatórias^(23)^, estimulando a proliferação e capacidade de migração da síntese de colágeno de fibroblastos; e melhora da drenagem linfática^(24)^.

De maneira geral, o histórico de uso da FBM na Fonoaudiologia é incipiente, diferentemente de outras profissões, tais como a Odontologia e a Fisioterapia, que possuem recomendações de uso mais definidas^(25)^. Além disso, há um número reduzido de publicações e de evidências externas robustas quanto à sua aplicação, especificamente, no âmbito do tecido e musculatura relacionados à laringe^(26)^. Em 17 de março de 2021, o Conselho Federal de Fonoaudiologia (CFFa) publicou a Resolução nº 606, sobre o uso do dispositivo de FBM por fonoaudiólogos, normatizando seu uso no exercício da atividade profissional. De acordo com tal Resolução, a FBM pode ser utilizada como dispositivo terapêutico, associado aos procedimentos clínicos fonoaudiológicos convencionais^(27)^.

A incorporação de uma nova estratégia ou conduta na área da saúde deve ser pautada na compreensão dos princípios subjacentes à estratégia e aos efeitos esperados (ou previamente verificados) em indivíduos que apresentam uma determinada condição de saúde^(28)^. Em condições ideais, o resultado de ensaios clínicos randomizados e estudos de revisão sistemática com metanálise devem ser considerados como as principais fontes de informação para a decisão clínica do profissional^(29)^. No entanto, a literatura disponível sobre o efeito da FBM na laringe ainda é escassa e com limitações metodológicas^(17,30-32)^, o que dificulta basear a tomada de decisão utilizando como fonte principal a literatura científica específica.

Nesse cenário, deve-se retomar que o conceito de prática baseada em evidência envolve três eixos principais, a saber: a evidência externa, advinda de pesquisas científicas; a opinião dos especialistas; e as preferências e valores dos pacientes^(33,34)^. Sendo assim, considerando que ainda não há evidência externa suficiente disponível quanto ao efeito imediato e de longo prazo da FBM na reabilitação e habilitação da voz, o uso atual desse dispositivo pode ser justificado por estratégias translacionais e analogias quanto aos efeitos da FBM em outros tecidos do corpo humano, pela percepção do clínico no ambiente terapêutico e pela avaliação/preferência do paciente quanto aos benefícios percebidos pelo uso da FBM.

Na ausência de evidências externas robustas, duas diretrizes importantes devem ser utilizadas pelos clínicos em sua tomada de decisão: os princípios biológicos e fisiopatológicos, e os princípios éticos^(35,36)^. Na da FBM na terapia e no treinamento vocal, fonoaudiólogos devem compensar a escassez de evidências científicas com um domínio dos princípios biológicos e fisiopatológicos, personalizando tratamentos para cada caso de disfonia ou de necessidades de profissionais da voz, sempre visando o uso seguro e eficaz da tecnologia para maximizar benefícios e reduzir riscos. Essencialmente, a prática deve ser guiada pela bioética, respeitando a autonomia do paciente através de comunicação transparente, exercendo a não maleficência com protocolos de segurança rigorosos, buscando a beneficência ao promover a recuperação vocal. Tais princípios éticos asseguram a responsabilidade dos clínicos na oferta da FBM como parte da terapia e treinamento vocal.

Nesse sentido, uma alternativa viável, na ausência de evidência externa disponível, é a realização de consensos entre especialistas da área com *expertise* na utilização de determinada estratégia terapêutica. O consenso pode se constituir em um ponto de partida para o direcionamento clínico dos critérios de utilização e um norteador para o desenvolvimento de pesquisas futuras, considerando-se que evocam o conhecimento biológico e fisiopatológico do clínico sobre a condição investigada, além dos princípios éticos relacionados à temática^(37-40)^.

Os métodos de consenso são cada vez mais utilizados como parte do desenvolvimento de diretrizes clínicas e de políticas de saúde, principalmente considerando o paradigma da prática baseada em evidência. Ele tende a impulsionar mudanças na prática clínica, uma vez que promove engajamento, consulta e validação entre os pares^(39,40)^. Além disso, os métodos de consenso possuem uma importância maior em temas cuja evidência existente é limitada, seja ela incipiente ou com baixa qualidade metodológica, como é o caso da FBM aplicada à habilitação e reabilitação vocal.

O método *Delphi* é uma das técnicas utilizadas para obter de forma sistemática o consenso sobre um tópico específico, como estratégias aplicadas em âmbito clínico, mas sem evidências científicas robustas. Nas pesquisas em saúde, este método começou a ser utilizado para definir áreas prioritárias de investigação e financiamento, tomadas de decisão e para incorporar tecnologias inovadoras e/ou dispositivos nos processos de habilitação e reabilitação de condições de saúde^(39)^.

Entre as principais vantagens do método *Delphi* estão o anonimato, a interação de diferentes especialistas, a possibilidade de repensar a opinião a partir de *feedbacks* controlados e a possibilidade de cumprir o objetivo principal que é chegar à resolutividade de um problema, ou à definição de um consenso sobre o tema específico^(38)^.

Dessa forma, considerando a escassez de literatura quanto ao uso da FBM na área de voz e a necessidade de ampliar o escopo da fundamentação para sustentar tal prática na área, a presente pesquisa tem por objetivo desenvolver um consenso com *experts* fonoaudiólogos acerca dos critérios de recomendação e uso da FBM no contexto de habilitação e reabilitação vocal, por meio do método *Delphi.*

## MÉTODO

### Desenho do estudo

Trata-se de um estudo transversal e descritivo para desenvolvimento de um consenso baseado na opinião de especialistas, por meio da técnica *Delphi.* Esta pesquisa foi submetida ao Comitê de Ética em Pesquisa com Seres Humanos da Instituição de origem e aprovado com o parecer de número: 3.998.709. Todos os voluntários do estudo tiveram acesso ao TCLE e concordaram em participar.

### Participantes

A seleção de um painel de especialistas é a parte fundamental do método *Delphi*. Em suma, apesar da falta de consenso sobre o que seja um especialista^(37)^, os membros do grupo devem ser comprometidos com o projeto, ter credibilidade e serem heterogêneos o suficiente para serem representativos de um conjunto de conhecimentos, competências e habilidades de alto nível sobre a área abordada ou problema discutido. Essas habilidades dependem principalmente do conhecimento dos especialistas^(41,42)^.

Para seleção dos participantes foram adotados os seguintes critérios de elegibilidade: ter no mínimo 10 anos de experiência clínica na área de voz, apresentar especialização na área de voz, mestrado e/ou doutorado em saúde e utilizar a FBM em sua prática clínica. Foram excluídos os fonoaudiólogos que não cumprissem tais critérios^(43)^.

Para o recrutamento, foram enviados e-mails para onze fonoaudiólogos especialistas em voz atuantes no Brasil. A lista desses profissionais foi fornecida por uma instituição que oferece cursos de pós-graduação tanto de especialização quanto de aperfeiçoamento, enquadrando-se como serviço de ensino-aprendizagem.

Dos onze profissionais contatados, três não utilizavam a FBM especificamente na área de voz, e um não utilizava esse dispositivo em sua prática clínica. Sendo assim, foram selecionados sete fonoaudiólogos, que cumpriam os critérios de elegibilidade. Não há uma definição quanto ao número de especialistas em um estudo de consenso^(39)^. A orientação principal é de que o número de participantes tenha uma relação com a magnitude do problema a ser investigado e que há pouca evidência empírica de que o número de participantes tem um efeito real na confiabilidade ou validade dos processos de consenso^(44)^. De maneira geral, o desenvolvimento de um consenso por meio do da técnica Delphi não requer o cálculo amostral, uma vez que essa técnica se baseia em uma série de rodadas de questionários/entrevistas anônimas e/ou iterativo para coletar opiniões e informações de um painel de especialistas. Ao contrário das pesquisas do tipo *web survey*, por exemplo, que demanda cálculo amostral que garanta a representatividade e generalização dos achados, a técnica Delphi não visa à representatividade probabilística da população, mas sim a obtenção do consenso entre os especialistas selecionados por sua experiência e conhecimento em uma determinada área^(45)^. Dessa forma, o aspecto mais importante é a qualificação do especialista participante^(45)^. Nesse sentido, considerando o objetivo do presente consenso e ausência de evidência empírica de que o número de especialistas afete no resultado e os critérios de elegibilidade definidos (que procuram garantir a qualificação do especialista), o número alcançado para essa pesquisa pode ser considerado válido.

A partir da confirmação da disponibilidade e dos critérios de elegibilidade, foi realizado um novo contato por e-mail, agendando uma data para a realização da primeira fase. As entrevistas com cada participante foram *on-line* e individuais com cada especialista. O perfil dos participantes pode ser conferido na Tabela 1.

**Tabela 1:** Caracterização profissional dos especialistas fonoaudiólogos que utilizam a FBM na área de Voz

**Variável**	**N**	**%**
**SEXO**		
**Feminino**	06	86
**Masculino**	1	14
**FAIXA ETÁRIA**		
**31-40 anos**	2	33
**41-50 anos**	4	45
	1	22
**51-60 anos**		
**TEMPO DE PROFISSÃO**		
**10-20 anos**	2	28,6
**20-30 anos**	5	71,4
**ESTADO ONDE EXERCE A FONOAUDIOLOGIA**		
**São Paulo**	2	28,57
**Minas Gerais**	1	14,28
**Bahia**	1	14,28
**Pernambuco**	1	14,28
**Paraná**	1	14,28
**Goiás**	1	14,28
**ESCOLARIDADE**		
**Especialização**	1	22
**Mestrado**	4	45
**Doutorado**	2	33
**ENSINO EM FONO**		
**Leciona em Pós-Graduação**	7	100
**Total**	7	100,0

Para eliminar o risco de influência devido à opinião de outros especialistas, todos os participantes permaneceram anônimos e desconhecidos uns dos outros durante todo o processo^(46)^.

### Procedimentos

Nesta pesquisa foi utilizada a técnica *Delphi,* por ser uma forma sistemática de obter consenso sobre determinado tema a partir de um painel de especialistas independentes^(47)^. A técnica *Delphi* é um processo estruturado para elencar, refinar e agregar as opiniões e percepções de um conjunto de pessoas (painel de especialistas) que possam contribuir de forma significativa, orientando as tomadas de decisões. A técnica Delphi é amplamente utilizada em estudos na área de saúde, incluindo estudos de consenso na área de Fonoaudiologia^(11,48-59)^. Quando a técnica é aplicada a temáticas cuja literatura científica baseada em dados empíricos é limitada, é realizada uma fase inicial de entrevistas individuais com especialistas para levantamento dos itens que serão analisados nas fases posteriores^(37,40,46,60)^.

Sendo assim, considerando que há uma limitação no número e na qualidade da evidência externa disponível para aplicação da FBM no contexto da terapia e do treinamento vocal, definiu-se a execução da técnica *Delphi* em duas rodadas: a primeira rodada correspondendo à realização de uma entrevista semiestruturada, permitindo que os especialistas elaborassem tópicos que considerassem importantes^(37,40,61)^, relacionados à FBM como alternativa complementar da intervenção fonoaudiológica na área de voz; a segunda rodada, relacionada ao julgamento de cada item pelos participantes com o objetivo de verificar a concordância entre os participantes e a seleção dos itens consensuais.

No processo terapêutico, os profissionais de saúde delineiam a intervenção levando em conta os alvos de tratamento, a seleção de ingredientes que serão utilizados para modificação de alvos e hipotetizam sobre os mecanismos de ação subjacentes aos ingredientes capazes de promover mudanças nos alvos^(62)^. A especificação dos ingredientes inclui a consideração sobre quantidade, modelagem, dosimetria, progressão e variabilidade na execução de um determinado procedimento com o paciente^(62)^. Dessa forma, nas duas fases da presente pesquisa procurou-se compreender a especificação do uso da FBM na habilitação e reabilitação vocal.

Para a elaboração dos itens a serem abordados na entrevista, realizou-se revisão da literatura, além da própria participação dos pesquisadores, a partir de sua experiência com o uso da FBM. Sendo assim, a versão inicial do instrumento de coleta de dados a ser utilizado na entrevista, incluiu 12 questões sobre o uso da FBM na área de voz (Quadro 1). Além disso, foram incluídas outras questões que contemplavam os dados sociodemográficos e o perfil profissional dos participantes.

**Quadro 1:** Questões da entrevista aprofundada FBM e a Voz Na fase 1 da Técnica Delphi

Questão 1 A FBM é um procedimento terapêutico para habilitação e reabilitação que vem sendo estudada e revisada no campo acadêmico, pois detém o poder da bioestimulação. De maneira geral, quais os benefícios e avanços a FBM trouxe para a Fonoaudiologia respectivamente para a área de voz?	Determinar os avanços da FBM especificamente para a área de Voz.
Questão 2 A terapia vocal tem como um dos objetivos modificar/gerenciar o comportamento vocal do indivíduo e desenvolver ajustes musculares funcionais e adaptados às demandas vocais do paciente. Você costuma utilizar a FBM em sua prática clínica na área de voz? Geralmente em que casos você faz mais uso da FBM?	Evidenciar a utilização da FBM na área de Voz.
Questão 3 A resolução do CFFa de nº 541, de 15 de março de 2019, dispõe sobre o uso do dispositivo de Laser de Baixa Intensidade como dispositivo terapêutico associado aos procedimentos clínicos fonoaudiológicos convencionais, podendo ser utilizado para fins fonoaudiológicos. Diante desta prerrogativa, a FBM é indicada para a área da voz? Você fez alguma formação/capacitação sobre a FBM em Fonoaudiologia com parâmetros clínicos na área de voz? Respectivamente, como seria a pré-prática (requisitos) para a recomendação e aplicação da FBM na área da voz?	Denotar a indicação da FBM para a prática fonoaudiológica, e os pré-requisitos para utilização na área de Voz.
Questão 4 O distúrbio de voz é multidimensional, pode ser decorrente de fatores comportamentais ou orgânicos, ou pela combinação destes. Representa qualquer dificuldade na emissão vocal que impeça a voz de cumprir seu objetivo que é a transmissão da mensagem verbal e emocional do indivíduo. Gostaria de considerar aqui três grandes grupos de disfonias: disfonia de origem comportamental sem lesão na laringe; disfonia de origem comportamental com lesão na laringe; e disfonias de origem orgânica. Considerando esses quadros, onde você tem feito o uso da FBM com mais frequência?	Elencar os casos em que o uso da FBM pode trazer mais resultados.
Questão 5 Alguns estudos evidenciam a fonoterapia como o tratamento de eleição para as disfonias, principalmente as relacionadas ao comportamento vocal. As etapas determinantes para uma repercussão positiva de tratamento devem contemplar orientação, conscientização e treinamento vocal com técnicas e exercícios específicos. Dessa forma, eu gostaria de fazer dois questionamentos relacionados ao uso da FBM na reabilitação vocal: a) em que momento do processo de reabilitação você utiliza a FBM: já no início do processo (primeiras sessões de acompanhamento do paciente), como um dispositivo complementar ao treinamento vocal (exercícios), ou como uma alternativa para os casos em que o treinamento vocal não está alcançando o resultado esperado? b) Em que momento da sessão você utiliza a FBM – antes, durante ou após a execução dos exercícios? Quais os critérios para essa escolha?	Evidenciar o momento de utilização da FBM no processo terapêutico.
Questão 6 No que concerne ao uso da FBM em indivíduos disfônicos, temos a experiência dos clínicos e a autorreferência de resultados positivos dos pacientes, porém não existem evidências científicas que sustentam a hipótese dos efeitos da FBM na área da voz. Quais as diretrizes clínicas você utiliza no que concerne à dose recomendada para pacientes com processos inflamatórios nas PPVV? Qual a quantidade de pontos por aplicação que são utilizadas em região laríngea? Como é verificado o efeito da FBM: solicita-se alguma tarefa específica antes e depois? Autorreferência do cliente? Acústica?	Inferir quais os parâmetros clínicos relacionados à dose, quantidade de pontos, e a verificação do efeito da FBM para indivíduos disfônicos.
Questão 7 As pregas vocais possuem uma geometria, histologia e viscoelasticidade distintas, com uma estrutura complexa, que inclui lâmina própria (em três camadas) e o músculo tireoaritenoideo, que sustentam sua estrutura biomecânica e a capacidade de fonação. No que se refere ao comprimento de onda, ao poder de ação e penetração da FBM, qual o comprimento de onda você mais utiliza na região de laringe?	Definir o comprimento de onda que considera mais adequado e efetivo da FBM em região laríngea.
Questão 8 A laringe é revestida por tecido de membrana mucosa, suas paredes são feitas de tecido conjuntivo, músculos e cartilagem. A cartilagem fornece suporte e mantém a elasticidade da laringe. Quanto às diretrizes clínicas da FBM, qual o método de irradiação mais utilizado em região laríngea e qual o local anatômico de aplicação mais utilizado em região laríngea? Por quê?	Evidenciar o método de irradiação da FBM em região laríngea e o local anatômico de aplicação.
Questão 9 No que se refere ao uso da FBM para aperfeiçoamento/condicionamento vocal para profissionais da voz, estudos denotam que os cromóforos sensibilizados pela FBM regulam componentes da cadeia respiratória, alterando seu potencial redox, promovendo ativação enzimática e o prolongamento da atividade bioquímica da fibra muscular. Quais as diretrizes clínicas você utiliza no que concerne à dose recomendada para pacientes de aperfeiçoamento/condicionamento vocal? Gostaria de considerar dois grupos de aperfeiçoamento/condicionamento vocal – voz falada e aperfeiçoamento/condicionamento – voz cantada. Considerando estes grupos, onde você tem feito o uso da FBM com mais frequência? Qual a quantidade de pontos por aplicação que são utilizadas em região laríngea? Como é verificado o efeito da biomodulação: solicita-se alguma tarefa específica antes e depois? Autorreferência do cliente? Acústica?	Elencar os casos em que o uso da FBM pode trazer mais resultados. Inferir quais os parâmetros clínicos relacionados à dose, quantidade de pontos, e a verificação do efeito da FBM para aperfeiçoamento/condicionamento vocal.
Questão 10 Em que momento do processo de habilitação você utiliza a FBM para indivíduos com alta performance vocal? Já no início do processo (primeiras sessões de acompanhamento do paciente), como um dispositivo complementar ao treinamento vocal (exercícios), ou como uma alternativa para os casos em que o treinamento vocal não está alcançando o resultado esperado? b) Em que momento da sessão você utiliza a FBM – antes, durante ou após a execução dos exercícios? Quais os critérios para essa escolha?	Delinear o momento de utilização da FBM no processo terapêutico.
Questão 11 Qual o método de irradiação e o local anatômico de aplicação da FBM você geralmente utiliza em região laríngea para aperfeiçoamento/condicionamento vocal? E por quê?	Identificar o método de irradiação da FBM em região laríngea e o local anatômico de aplicação.
Questão 12 A técnica ILIB modificado ou laserterapia sistêmica consiste na aplicação não invasiva, contínua e indireta de laser terapêutico vermelho (660nm) na região da artéria radial, de forma contínua. Esta absorção pelo sangue acarreta um aumento no metabolismo e na síntese da principal proteína fisiológica reguladora do sistema oxidativo corpóreo (superóxido dismutase). Essa enzima inibe a ação das espécies reativas de oxigênio (EROs), leva à proteção das células contra mutações e envelhecimento, combatendo assim os radicais livres tão prejudiciais à saúde. Você utiliza a técnica ILIB em pacientes disfônicos? Ou para condicionamento/aperfeiçoamento vocal?	Verificar a utilização da técnica ILIB ou laserterapia sistêmica na área de Voz.

**Legenda**: FBM = fotobiomodulação; ILIB = Intravascular Laser Irradiation of Blood

As respostas ao questionário foram abertas, para obter informações mais amplas a respeito das percepções acerca do tema e de escopo da atuação fonoaudiológica em habilitação e reabilitação vocal^(63)^.

#### Primeira rodada

O objetivo desta fase foi coletar as opiniões, as experiências e a prática clínica com a utilização da FBM na área de voz. Os especialistas foram convidados a participar de uma entrevista aprofundada sobre o uso da FBM na área de Voz, baseada nas questões descritas no Quadro 1. A entrevista teve duração entre 40-60 minutos e foi realizada por videoconferência, via plataforma Zoom. Elas foram conduzidas pelo pesquisador principal e realizadas individualmente com cada participante. O material das entrevistas foi registrado em áudio e vídeo para posterior consulta e transcrição.

Após a transcrição de todo o material da entrevista obtido com os sete entrevistados, o conteúdo foi sintetizado e transformado em 55 frases afirmativas. Tais frases foram revisadas por dois outros pesquisadores envolvidos neste trabalho, ambos especialistas em voz e com formação em FBM. Os revisores verificaram a correspondência entre as frases afirmativas e o conteúdo original transcrito das entrevistas. Este procedimento teve por objetivo reduzir o risco de viés. Após a revisão e respectivas reformulações, a versão final das frases pode ser conferida no Quadro 2.

**Quadro 2:** Afirmações acerca da fotobiomodulação aplicada à área de voz

**ITENS**
1. – Eu considero que a fotobiomodulação (FBM) é indicada para utilização na área de voz.
2. – Realizo a aplicação da FBM no início do processo de reabilitação vocal.
3. – Utilizo a FBM como um dispositivo complementar ao treinamento vocal.
4. - Realizo aplicação da FBM como uma alternativa complementar apenas para os casos em que tenho limitações no resultado com a utilização da terapia vocal convencional.
5. - Eu considero que ter uma formação/capacitação sobre FBM é imprescindível para o seu uso efetivo e aplicação clínica na área de voz.
6. - Eu utilizo a FBM em profissionais da voz sem disfonia.
7. - Eu utilizo a FBM apenas nos casos de aperfeiçoamento vocal.
8. - Eu utilizo a FBM na reabilitação vocal de pacientes disfônicos.
9. - Eu utilizo a FBM na área de Voz para melhorar o desempenho muscular do cliente/paciente.
10. - Eu utilizo a FBM na área de Voz para a modulação da inflamação.
11. - Eu utilizo a FBM em profissionais da voz para melhorar o desempenho muscular.
12. - Eu utilizo a FBM em profissionais da voz para melhorar a recuperação muscular.
13. - Eu utilizo a FBM em profissionais da voz para diminuir os sintomas de fadiga vocal.
14. - Eu utilizo a FBM nos casos de pacientes com disfonias por tensão muscular.
15. - Eu utilizo a FBM nos casos de pacientes com paralisias laríngeas.
16. - Eu utilizo a FBM nos casos de pacientes com disfonia comportamental sem lesão na laringe.
17. - Eu utilizo a FBM nos casos de pacientes com disfonia comportamental com lesão na laringe.
18. - Eu utilizo a FBM nos casos de pacientes com disfonia de origem orgânica
19. - Eu utilizo a FBM nos casos de pacientes com disfonia de origem neurológica.
20. - Eu utilizo a FBM nos casos de pacientes com disfonia por sequelas de câncer de cabeça e pescoço.
21. - Eu utilizo a FBM nos casos de pacientes com disfonia decorrentes de laringite por refluxo laringofaríngeo.
22. - Eu utilizo a FBM em região laríngea antes da execução dos exercícios vocais.
23. - Eu utilizo a FBM em região laríngea durante a execução dos exercícios vocais.
24. - Eu utilizo a FBM em região laríngea após a execução dos exercícios vocais.
25. - Eu utilizo o comprimento de onda infravermelho em região laríngea em pacientes com processos inflamatórios nas pregas vocais.
26. - Eu utilizo o contato pontual como método de irradiação em região laríngea em pacientes com processos inflamatórios nas pregas vocais.
27. - Eu aplico a FBM na lâmina da cartilagem tireoide em pacientes com processos inflamatórios nas pregas vocais.
28. - Eu aplico a FBM na comissura anterior, e na lâmina da cartilagem tireoide em pacientes com processos inflamatórios nas pregas vocais.
29. - Eu aplico 4J (Joules) por ponto em região laríngea em pacientes com processos inflamatórios nas pregas vocais.
30. - Eu aplico de 4-6J (Joules) por ponto em região laríngea nos pacientes com processos inflamatórios nas pregas vocais.
31. - Eu aplico 6J (Joules) por ponto em região laríngea nos pacientes com processos inflamatórios nas pregas vocais.
32. - Eu aplico a FBM em 3 pontos nas hemilaringes de pacientes com processos inflamatórios nas pregas vocais.
33. - Eu aplico a FBM em 4 pontos nas hemilaringes de pacientes com processos inflamatórios nas pregas vocais.
34. - Eu aplico a FBM em 5 pontos nas hemilaringes de pacientes com processos inflamatórios nas pregas vocais.
35. - Eu solicito uma tarefa vocal específica antes e após aplicação, para verificar o efeito da FBM na voz.
36. - Eu utilizo a autorreferência do paciente/cliente para verificar o efeito da FBM na produção vocal.
37. - Eu utilizo a análise acústica antes e após a aplicação da FBM para verificar o seu efeito na voz.
38. - Eu utilizo a FBM como dispositivo complementar para o condicionamento vocal em profissionais da voz falada.
39. - Eu utilizo a FBM como dispositivo complementar para o condicionamento vocal em profissionais da voz cantada.
40. - Utilizo doses menores que 4J (Joules) quando o meu objetivo é melhorar o condicionamento vocal em profissionais da voz.
41. - Utilizo doses entre 4-6J (Joules) quando o meu objetivo é melhorar o condicionamento vocal em profissionais da voz.
42. - Utilizo doses entre 6-9J (Joules) quando o meu objetivo é melhorar o condicionamento vocal em profissionais da voz.
43. - Utilizo o comprimento de onda infravermelho em região laríngea quando o meu objetivo é melhorar o condicionamento vocal do cliente/paciente.
44. - Utilizo o contato pontual como método de irradiação em região laríngea quando o meu objetivo é melhorar o condicionamento vocal do cliente/paciente.
45. - Aplico a FBM em 3 pontos nas hemilaringes quando o meu objetivo é melhorar o condicionamento vocal do cliente/paciente.
46. - Aplico a FBM em 4 pontos nas hemilaringes quando o meu objetivo é melhorar o condicionamento vocal do cliente/paciente.
47. - Aplico a FBM em 5 pontos nas hemilaringes quando o meu objetivo é melhorar o condicionamento vocal do cliente/paciente.
48. - Utilizo a FBM em região laríngea antes da execução dos exercícios vocais quando o meu objetivo é condicionamento vocal de profissionais da voz.
49. - Utilizo a FBM em região laríngea durante a execução dos exercícios vocais quando o meu objetivo é condicionamento vocal de profissionais da voz.
50. - Utilizo a FBM em região laríngea após a execução dos exercícios vocais quando o meu objetivo é condicionamento vocal de profissionais da voz.
51. - Utilizo o contato pontual como método de irradiação em região laríngea quando o meu objetivo é condicionamento vocal de profissionais da voz.
52. - Aplico a FBM na lâmina da cartilagem tireoide quando o meu objetivo é condicionamento vocal de profissionais da voz.
53. - Aplico a FBM na comissura anterior, e na quilha da cartilagem tireoide em região laríngea quando o meu objetivo é condicionamento vocal de profissionais da voz.
54. - Eu utilizo a técnica ILIB em pacientes disfônicos.
55 - Eu utilizo a técnica ILIB em clientes cujo objetivo é condicionamento/aperfeiçoamento vocal.

#### Segunda rodada

O objetivo da segunda rodada foi obter a opinião dos especialistas acerca dos parâmetros dosimétricos e critérios de uso da FBM em voz. Sendo assim, os participantes foram convidados a responder um questionário via *Google Forms*.

As sentenças obtidas e formuladas ao final da primeira rodada foram apresentadas uma a uma para os participantes, que deveriam responder quanto ao seu grau de concordância com cada afirmativa. Para tanto, foi utilizada uma escala Likert de cinco pontos, com respostas que variavam de “concordo totalmente” (1) a “discordo totalmente” (5) (Figura 1). Essa escala foi utilizada por ser de fácil compreensão e permitir a análise das respostas estatisticamente por meio do coeficiente de validade de conteúdo (CVC)^(40,46)^. Além disso, os participantes poderiam fornecer comentários sobre cada item para qualificar sua resposta e ajudar na reformulação da frase declarativa para iterações futuras^(37,47)^.

**Figura 1:** Modelo das frases e escala Likert apresentadas na segunda rodada

*Frase deve ser obrigatoriamente respondida

Legenda: N/A = não se aplica

Todos os participantes receberam o *link* para acessar o Termo de Consentimento Livre e Esclarecido (TCLE), as orientações para realização desta etapa e o instrumento com os 55 itens da pesquisa. Inicialmente, o participante era convidado a ler integralmente o TCLE e confirmar ou não a sua participação na pesquisa. Em caso de aceite, o participante era conduzido à primeira seção, composta por orientações de preenchimento do instrumento, e às seções seguintes, que continham as afirmações acerca da FBM na prática clínica de voz.

Após responder todas as questões, o especialista acionava o comando “enviar” e o procedimento de coleta era finalizado. O tempo para concluir o formulário foi estimado em cerca de 15 minutos. O instrumento ficou disponível para respostas durante oito semanas. Os dados obtidos nesta etapa foram extraídos da plataforma *Google Forms* para uma planilha do *Microsoft Excel,* com o objetivo de realização da análise estatística.

Nesta pesquisa, optou-se pela definição de duas rodadas para a obtenção dos itens consensuais^(61,64,65)^. A recomendação para duas rodadas baseia-se no fato de que os participantes podem se cansar de responder os mesmos itens e tendem a se inclinar a mudar a resposta simplesmente para encerrar a sua participação. Dessa forma, ao final da segunda rodada os dados foram tabulados e submetidos à análise estatística para verificação da concordância e definição do consenso.

### Análise estatística

Foi utilizado o CVC como principal critério para definição do consenso em cada item. O CVC investiga o grau de concordância entre os juízes na avaliação de cada item do questionário^(66-68)^.

Para o cálculo do CVC foram obtidas as médias, o total e o valor de erro das notas da escala Likert (1 a 5 pontos para cada item) de cada frase do questionário. Para o cálculo final foi utilizado o CVC de cada item subtraído pelo constante da fórmula ou cálculo do erro. O método utilizado para a obtenção do coeficiente é apresentado^(69,70)^ no Quadro 3.

**Quadro 3:** Método utilizado para o cálculo do CVC

Fórmula do CVC
$1{-}_{i}\frac{\left(\frac{\sum_{i}}{n}\right)}{\max}2{-}_{i}{\left(\frac{}{n}\right)}^{n}3{-}_{iii}$


Nesta pesquisa, foi adotada a seguinte classificação^(71)^ para a interpretação dos valores do CVC: excelente (CVC ≥ 0,78), bom (0,60 ≥ CVC ≤ 0,77) e ruim (CVC ≥ 0,59). Considerando-se que o objetivo desta pesquisa é desenvolver um consenso acerca do uso da FBM na terapia e treinamento vocal, foram considerados aceitáveis e consensuais apenas os itens que apresentaram um CVC acima de 0,75^(67,72)^. Os dados foram analisados por meio do *software* SPSS versão 22.

## RESULTADOS

A partir das análises feitas, observou-se consenso em 34 itens (CVC ≥ 0,75) (Tabela 2).

**Tabela 2:** Coeficiente de Validade de Conteúdo dos itens relacionados ao uso da fotobiomodulação na habilitação e reabilitação vocal

**ITEM**	**CVC**
1. Eu considero que a fotobiomodulação (FBM) é indicada para utilização na área de voz.	1,00*
2. Realizo a aplicação da FBM no início do processo de reabilitação vocal.	0,86*
3. Utilizo a FBM como um dispositivo complementar ao treinamento vocal.	1,00*
4. Realizo aplicação da FBM como uma alternativa complementar apenas para os casos em que tenho limitações no resultado com a utilização da terapia vocal convencional.	0,52
5. Eu considero que ter uma formação/capacitação sobre FBM é imprescindível para o seu uso efetivo e aplicação clínica na área de voz.	1,00*
6. Eu utilizo a FBM em profissionais da voz sem disfonia.	1,00*
7. Eu utilizo a FBM apenas nos casos de aperfeiçoamento vocal.	0,71
8. Eu utilizo a FBM na reabilitação vocal de pacientes disfônicos.	0,86*
9. Eu utilizo a FBM na área de Voz para melhorar o desempenho muscular do cliente/paciente.	0,95*
10. Eu utilizo a FBM na área de Voz para a modulação da inflamação.	0,71
11. Eu utilizo a FBM em profissionais da voz para melhorar o desempenho muscular.	0,90*
12. Eu utilizo a FBM em profissionais da voz para melhorar a recuperação muscular.	0,90*
13. Eu utilizo a FBM em profissionais da voz para diminuir os sintomas de fadiga vocal.	1,00*
14. Eu utilizo a FBM nos casos de pacientes com disfonias por tensão muscular.	0,71
15. Eu utilizo a FBM nos casos de pacientes com paralisias laríngeas.	0,62
16. Eu utilizo a FBM nos casos de pacientes com disfonia comportamental sem lesão na laringe.	0,81*
17. Eu utilizo a FBM nos casos de pacientes com disfonia comportamental com lesão na laringe.	0,52
18. Eu utilizo a FBM nos casos de pacientes com disfonia de origem orgânica	0,57
19. Eu utilizo a FBM nos casos de pacientes com disfonia de origem neurológica.	0,48
20. Eu utilizo a FBM nos casos de pacientes com disfonia por sequelas de câncer de cabeça e pescoço.	0,43
21. Eu utilizo a FBM nos casos de pacientes com disfonia decorrentes de laringite por refluxo laringofaríngeo.	0,71
22. Eu utilizo a FBM em região laríngea antes da execução dos exercícios vocais.	1,00*
23. Eu utilizo a FBM em região laríngea durante a execução dos exercícios vocais.	0,48
24. Eu utilizo a FBM em região laríngea após a execução dos exercícios vocais.	0,81*
25. Eu utilizo o comprimento de onda infravermelho em região laríngea em pacientes com processos inflamatórios nas pregas vocais.	0,86*
26. Eu utilizo o contato pontual como método de irradiação em região laríngea em pacientes com processos inflamatórios nas pregas vocais.	0,95*
27. Eu aplico a FBM na lâmina da cartilagem tireóide em pacientes com processos inflamatórios nas pregas vocais.	0,81*
28. Eu aplico a FBM na comissura anterior, e na lâmina da cartilagem tireóide em pacientes com processos inflamatórios nas pregas vocais.	0,86*
29. Eu aplico 4J (Joules) por ponto em região laríngea em pacientes com processos inflamatórios nas pregas vocais.	0,76*
30. Eu Aplico de 4-6J (Joules) por ponto em região laríngea nos pacientes com processos inflamatórios nas pregas vocais.	0,86*
31. Eu aplico 6J (Joules) por ponto em região laríngea nos pacientes com processos inflamatórios nas pregas vocais.	0,95*
32. Eu aplico a FBM em 3 pontos nas hemilaringes de pacientes com processos inflamatórios nas pregas vocais.	0,62
33. Eu aplico a FBM em 4 pontos nas hemilaringes de pacientes com processos inflamatórios nas pregas vocais.	0,71
34. Eu aplico a FBM em 5 pontos nas hemilaringes de pacientes com processos inflamatórios nas pregas vocais.	0,43
35. Eu solicito uma tarefa vocal específica antes e após aplicação, para verificar o efeito da FBM na voz.	1,00*
36. Eu utilizo a autorreferência do paciente/cliente para verificar o efeito da FBM na produção vocal.	1,00*
37. Eu utilizo a análise acústica antes e após a aplicação da FBM para verificar o seu efeito na voz.	0,71
38. Eu utilizo a FBM como dispositivo complementar para o condicionamento vocal em profissionais da voz falada.	1,00*
39. Eu utilizo a FBM como dispositivo complementar para o condicionamento vocal em profissionais da voz cantada.	0,95*
40. Utilizo doses menores que 4J (Joules) quando o meu objetivo é melhorar o condicionamento vocal em profissionais da voz.	0,33
41. Utilizo doses entre 4-6J (Joules) quando o meu objetivo é melhorar o condicionamento vocal em profissionais da voz.	1,00*
42. Utilizo doses entre 6-9J (Joules) quando o meu objetivo é melhorar o condicionamento vocal em profissionais da voz.	0,95*
43. Utilizo o comprimento de onda infravermelho em região laríngea quando o meu objetivo é melhorar o condicionamento vocal do cliente/paciente.	0,81*
44. Utilizo o contato pontual como método de irradiação em região laríngea quando o meu objetivo é melhorar o condicionamento vocal do cliente/paciente.	0,90*
45. Aplico a FBM em 3 pontos nas hemilaringes quando o meu objetivo é melhorar o condicionamento vocal do cliente/paciente.	0,76*
46. Aplico a FBM em 4 pontos nas hemilaringes quando o meu objetivo é melhorar o condicionamento vocal do cliente/paciente.	0,76*
47. Aplico a FBM em 5 pontos nas hemilaringes quando o meu objetivo é melhorar o condicionamento vocal do cliente/paciente.	0,62
48. Utilizo a FBM em região laríngea antes da execução dos exercícios vocais quando o meu objetivo é condicionamento vocal de profissionais da voz.	0,95*
49. Utilizo a FBM em região laríngea durante a execução dos exercícios vocais quando o meu objetivo é condicionamento vocal de profissionais da voz.	0,43
50. Utilizo a FBM em região laríngea após a execução dos exercícios vocais quando o meu objetivo é condicionamento vocal de profissionais da voz.	0,33
51. Utilizo o contato pontual como método de irradiação em região laríngea quando o meu objetivo é condicionamento vocal de profissionais da voz.	0,90*
52. Aplico a FBM na lâmina da cartilagem tireoide quando o meu objetivo é condicionamento vocal de profissionais da voz.	0,90*
53. Aplico a FBM na comissura anterior, e na quilha da cartilagem tireoide em região laríngea quando o meu objetivo é condicionamento vocal de profissionais da voz.	0,81*
54. Eu utilizo a técnica ILIB em pacientes disfônicos.	0,62
55. Eu utilizo a técnica ILIB em clientes cujo objetivo é condicionamento/aperfeiçoamento vocal.	0,71
**Total**	0,78*

* Itens com CVC > 0,75

Legenda: CVC = Coeficiente de validade de conteúdo

Observou-se que 31 itens obtiveram CVC excelente (≥ 0,78), 14 itens com bom CVC (0,60 ≥ IVC ≤ 0,77) e 10 itens com CVC ruim (≥ 0,59). O CVC total foi considerado excelente, com valor igual a 0,78.

## DISCUSSÃO

O objetivo deste estudo foi desenvolver um consenso acerca das diretrizes clínicas para o uso da FBM na habilitação e reabilitação vocal que dê suporte ao exercício profissional do fonoaudiólogo nesse campo e forneça indicadores para futuras pesquisas na área. De maneira geral, o resultado da aplicação dos métodos de consenso está relacionado à melhora da tomada de decisão, desenvolvimento de critérios para determinada prática e fornecimento de balizadores na ausência de evidência externa suficiente ou incerteza quanto ao efeito e efetividade de tal prática para uma condição específica^(73)^.

Nesta pesquisa, sete especialistas participaram das duas rodadas realizadas, sem perda de participantes entre uma rodada e outra. O comprometimento do painel de especialistas é fundamental neste tipo de pesquisa e pode refletir o nível de interesse pela temática^(74)^, especificamente no que diz respeito à fundamentação da especificação terapêutica da FBM como estratégia complementar no processo de habilitação e reabilitação vocal.

Na presente pesquisa, houve consenso em 34 itens relacionados ao uso da FBM na área de voz. Didaticamente, agrupamos os itens do consenso em categorias para facilitar o fluxo das informações na discussão, incluindo: características gerais sobre uso da FBM em voz; seleção dos pacientes ou clientes para aplicação da FBM; alvos terapêuticos com o uso da FBM; momento da irradiação; dose de FBM realizada; local de aplicação da FBM; modo e pontos de aplicação da FBM; comprimento de onda utilizado; e medidas de efeito após o uso da FBM.

### Características gerais sobre o uso da FBM em voz

Seis itens (1, 3, 4, 5, 38 e 39) abordavam questões relacionadas às características gerais sobre o uso da FBM em voz. Entre esses, houve consenso em cinco itens, sendo a exceção, o item de número quatro.

Os especialistas consideram que a FBM é indicada para utilização na área de voz (item 1) e a utilizam como um dispositivo complementar ao treinamento vocal (itens 3, 38 e 39). Dessa forma, observa-se que, embora não haja evidência externa disponível e com elevado nível de qualidade quanto ao uso da FBM na área de voz, os especialistas têm utilizado esse dispositivo no campo da terapia e treinamento vocal. De maneira geral, os fonoaudiólogos têm se apoiado nos estudos com ciências básicas e por ilação, estabelecendo-se inferências lógicas, a partir de resultados de pesquisas sobre sua aplicação em outras estruturas da região de cabeça e pescoço, ou em conclusões baseadas no raciocínio clínico a partir dos efeitos esperados da irradiação nos tecidos corporais.

Nesse sentido, considerando-se que a prática baseada em evidência se baseia no tripé da evidência externa, opinião dos especialistas e preferências dos pacientes, o uso da FBM na área de voz parece estar baseado na opinião dos especialistas e nas preferências dos pacientes, na ausência de evidência externa disponível. Além disso, destaca-se que a Resolução nº 606 do CFFa regulamenta a possibilidade de uso da FBM pelo fonoaudiólogo no campo da voz humana, sempre associado como dispositivo complementar no processo de terapia ou treinamento vocal^(75)^.

A expertise clínica e as preferências do paciente ou cliente são elementos fundamentais para a personalização do tratamento. Nesse sentido, enquanto novas pesquisas são desenvolvidas para fornecer uma base de evidências mais robusta (que suporte ou não o uso da FBM em diferentes contextos da área de voz), a experiência clínica e a opinião do paciente ou cliente são aspectos valiosos para a tomada de decisão.

Os fonoaudiólogos participantes do estudo obtiveram consenso quanto à necessidade de capacitação específica e complementar para o uso desse dispositivo na clínica vocal (item 5). Tal achado está em consonância com a Resolução mencionada, a qual preconiza que o fonoaudiólogo só poderá utilizar o dispositivo terapêutico da FBM quando possuir capacitação específica e adequada, estando sujeito à responsabilidade legal em casos de imperícia, negligência e imprudência. A existência de uma regulamentação específica é um indicador da legitimidade da prática e da necessidade de garantir que os profissionais estejam adequadamente treinados.

Os especialistas não apresentaram concordância no que se refere à aplicação da FBM como uma alternativa complementar nos casos em que há apenas limitações no resultado com a terapia convencional (Item 4). A ausência de consenso nesse item pode refletir o desafio inerente à incorporação de novas tecnologias e dispositivos em práticas clínicas estabelecidas, especialmente em um campo em que existe uma ampla variedade de ingredientes para alcançar os mesmos alvos terapêuticos.

A falta de consenso sobre o uso da FBM como uma estratégia complementar pode estar relacionada à valorização da terapia vocal tradicional como o padrão-ouro, cuja eficácia e segurança são amplamente reconhecidas e apoiadas por uma vasta literatura científica. Além disso, a prudência na recomendação de novos dispositivos é uma característica desejável na prática baseada em evidências, garantindo que novas abordagens sejam adotadas com base em resultados comprovados e não apenas por novidade ou tendência.

Por outro lado, a não concordância quanto ao uso limitado da FBM pode indicar uma abertura para explorar seu potencial mais amplamente, não apenas como um dispositivo a ser utilizado nas situações de limitação da terapia convencional, mas como um ingrediente a ser associado à terapia convencional.

De igual modo, não houve consenso entre os especialistas a respeito do uso da ILIB (*Intravascular Laser Irradiation of Blood* - Fotobiomodulação Sistêmica Vascular), seja para o tratamento de processos inflamatórios nas pregas vocais, seja para o trabalho com o condicionamento/aperfeiçoamento vocal (itens 54 e 55).

A técnica ILIB consiste na irradiação vermelha (mais utilizada) ou infravermelha intravascular do sangue. Na Fonoaudiologia é executada de forma não-invasiva e transdérmica, por meio de um feixe luminoso posicionado no pulso sobre a artéria radial ou carótida. Seu objetivo é irradiar a corrente sanguínea e, com isso, estimular a ação do organismo como um todo^(76)^. Não existem estudos que evidenciam o principal efeito desta para habilitação e reabilitação em voz^(76)^. Dessa forma, a falta de consenso pode estar relacionada à ausência de evidências científicas robustas quanto ao uso para o objetivo específico de aperfeiçoamento ou reabilitação vocal.

Em síntese, o resultado do consenso nessa subseção sobre as características gerais sobre o uso da FBM em voz reforça a necessidade de uma abordagem mais matizada para a adoção de dispositivos complementares à terapia vocal convencional. Deve-se considerar tanto o respeito pelas metodologias tradicionais quanto a abertura para a inovação. Uma consideração cuidadosa sobre como e quando integrar novas abordagens pode levar a um paradigma de tratamento mais holístico e personalizado, onde a FBM não é vista como um último recurso, mas como uma parte valiosa de um repertório terapêutico diversificado.

O consenso sobre o uso potencial da FBM na área de voz como dispositivo complementar à terapia convencional, pode destacar a natureza multimodal da terapia e treinamento vocal, onde diferentes ingredientes podem ser utilizados para alcançar o melhor resultado possível para o paciente ou cliente.

### Seleção dos pacientes ou clientes para aplicação da FBM

Dez itens (6, 8, 14, 15, 16-21) abordavam questões quanto aos critérios de eleição dos potenciais pacientes ou clientes para uso da FBM em voz, três consensuais (itens 6, 8 e 16).

Os especialistas concordaram em utilizar este dispositivo no treinamento vocal de profissionais da voz sem disfonia e no processo de reabilitação de pacientes disfônicos. A justificativa para tal uso pode estar associada aos seus principais efeitos. Estudos na área de fisioterapia com ratos e humanos, bem como consenso em motricidade orofacial ratificam que a radiação no espectro do visível e infravermelho próximo atua como um agente biomodulador, capaz de promover efeitos anti-inflamatórios e analgésicos, por indução de respostas celulares e sistêmicas. Levando isso em conta, formula-se a hipótese, que pode ser a base do raciocínio que permitiu o consenso desses itens, a respeito da possível melhora do desempenho vocal do indivíduo, principalmente, quando submetido a uma alta demanda de voz^(22,26,77-79)^.

Mais especificamente, a respeito do uso desse dispositivo em pacientes disfônicos, houve concordância quanto ao seu emprego em pacientes com disfonia comportamental sem lesão laríngea. A disfonia comportamental é multifatorial, contudo, tem como principal etiologia uso inadequado vocal, em conjunto à exposição a fatores de riscos para o distúrbio da voz^(80)^. Ela é caracterizada pelo aumento de atividade muscular com tensão e esforço de musculatura intrínseca, e por vezes, recrutamento de musculatura intrínseca da laringe durante a fonação. Possivelmente, essa concordância entre os especialistas tem associação com o aumento de ATP muscular e, por conseguinte, redução da fadiga muscular atribuída à FBM^(78,81)^. Estudo visando investigar o efeito da FBM na atenuação da fadiga vocal, após tarefa de sobrecarga concluiu que a utilização da FBM é potencialmente eficaz na redução fadiga e pode ter ampla relevância clínica para as populações com distúrbios de voz ou ocupações com alta demanda de uso da voz^(17)^.

Não foi obtida concordância entre os especialistas no que se refere ao uso da FBM com os objetivos de modulação da inflamação, em disfonias por tensão muscular, disfonias comportamentais com lesão na laringe; em pacientes com paralisias laríngeas, ou em casos de laringite por refluxo laringofaríngeo, disfonias orgânicas e neurológicas e em indivíduos com sequela de câncer de cabeça e pescoço. Paralelamente à ausência de evidências científicas robustas que embasariam a aplicação da FBM nesses casos, autores pontuam que pode haver restrições no uso da FBM em casos pré-cancerígenos e/ou cancerígenos, por induzir a proliferação de células cancerígenas^(82-87)^.

A não concordância em relação ao uso da FBM em uma variedade de outras condições vocais, incluindo disfonias por tensão muscular com lesão na laringe, paralisias laríngeas, laringite por refluxo, disfonias orgânicas e neurológicas, e em indivíduos com sequelas de câncer de cabeça e pescoço, sinaliza cautela entre os especialistas. Isso pode ser atribuído à falta de evidências robustas e à preocupação com a segurança, particularmente em relação ao potencial de proliferação de células cancerígenas. A posição conservadora dos especialistas pode refletir uma responsabilidade ética em não expor pacientes a tratamentos cujos benefícios e segurança ainda não estejam claramente estabelecidos na literatura científica. Além disso, a falta de consenso quanto a condições vocais específicas pode denotar que os especialistas ainda não tenham hipóteses e critérios bem definidos sobre como tais condições respondem, especificamente, à FBM.

A falta de consenso entre especialistas sobre o uso da FBM apenas para o aperfeiçoamento vocal levanta questões pertinentes sobre a aplicabilidade e os limites de intervenções com o uso desse dispositivo. Do ponto de vista clínico, a cautela dos especialistas pode ser interpretada como um reflexo da natureza ainda emergente da FBM dentro do campo do treinamento vocal. Os especialistas podem estar considerando que a FBM, em sua essência, é um dispositivo com indicações terapêuticas específicas e não um dispositivo com o foco direto no aprimoramento vocal. Do ponto de vista pragmático, a incorporação de um novo dispositivo em práticas estabelecidas exige treinamento, custos e uma curva de aprendizado que talvez não se justifiquem para o aperfeiçoamento vocal, especialmente se houver métodos menos invasivos e mais tradicionais disponíveis com resultados comprovados.

Adicionalmente, o uso da palavra “apenas” pode ter conotado uma limitação que não ressoa bem com a abordagem de treinamento mais ampla dos especialistas, que pode favorecer uma abordagem mais integrativa, considerando a FBM como parte de um conjunto maior de estratégias para o aperfeiçoamento vocal.

A não concordância também pode refletir uma hesitação em aprovar a FBM para aperfeiçoamento vocal devido à ausência de evidências robustas nesse contexto específico, assim como tal escassez em toda a área de voz. Portanto, a discussão em torno do uso da FBM apenas para aperfeiçoamento vocal permanece uma área de debate ativo. Em última análise, a decisão de utilizar a FBM para aperfeiçoamento vocal deve ser tomada com base em uma consideração cuidadosa das implicações clínicas, éticas e práticas, e sempre com um olhar atento à evolução da pesquisa e das diretrizes profissionais.

Essa falta de consenso no campo do aperfeiçoamento vocal pode indicar que os especialistas têm um maior entendimento ou uma preferência quanto ao uso desse dispositivo com foco primariamente terapêutico, e não necessariamente como uma ferramenta direcionada ao aprimoramento vocal. Essa distinção é crucial, pois implica uma reflexão sobre as indicações e limitações da FBM.

Portanto, a discussão sobre a aplicabilidade da FBM para aperfeiçoamento vocal permanece aberta e ativa, sublinhando a necessidade de uma avaliação cuidadosa das implicações clínicas, éticas e práticas. A decisão de empregar a FBM para esse fim deve ser fundamentada em uma análise criteriosa da literatura existente, bem como na evolução contínua da pesquisa e das diretrizes profissionais, garantindo que as práticas adotadas estejam alinhadas com as melhores evidências disponíveis e com os princípios de cuidado ao paciente.

Por outro lado, um estudo realizado com 148 fonoaudiólogos brasileiros com atuação na área de voz, revelou que eles usam esse dispositivo em casos de disfonia comportamental, independentemente da presença de lesões nas pregas vocais, assim como em indivíduos com uso profissional da voz em disfonia neurológica^(10)^. Dessa forma, observa-se que os especialistas selecionados para a presente pesquisa apresentaram uma postura mais conservadora em relação aos pacientes/clientes elegíveis para o uso da FBM.

### Alvos terapêuticos com o uso da FBM

Dos seis pontos que tratam dos alvos terapêuticos no uso da FBM (itens 7, 9, 10, 11-13), quatro obtiveram consenso (itens 9, 11-13). Eles dizem respeito à melhora do desempenho e recuperação muscular e diminuição dos sintomas de fadiga vocal do cliente/paciente, incluindo profissionais da voz. Não houve consenso a respeito do uso deste dispositivo para modulação da inflamação.

Os achados em questão abordam um tema central e atual na área da fonoaudiologia e terapia vocal: a utilização da FBM como um dispositivo para melhorar a performance vocal e auxiliar na recuperação muscular, bem como na diminuição dos sintomas de fadiga vocal. Estes aspectos são de particular interesse para profissionais da voz, cuja carreira depende intrinsecamente da saúde e funcionalidade da produção vocal.

A melhora do desempenho vocal e a recuperação muscular são objetivos fundamentais na reabilitação vocal e no treinamento de profissionais da voz. A FBM, a partir dos estudos que a fundamentam, é apresentada como uma intervenção promissora devido aos seus potenciais efeitos biomoduladores. Na área da ciência do esporte, existem evidências que o uso da FBM está relacionado ao aumento da performance muscular e diminuição da fadiga, por conta de seu mecanismo de ação^(14-17,31,88-91)^. Como resultados desses estudos^(14-17,92-94)^, a aceleração das reações da cadeia oxidativa em nível mitocondrial, o aumento da microcirculação, a melhora da drenagem linfática, da proliferação e mobilidade das células satélites, acelerando a síntese de colágeno e reduzindo a resposta inflamatória, além de cicatrização de tecidos de forma efetiva^(14-17,31,88-91)^. Único estudo de intervenção na área de voz que investigou efeito da FBM na atenuação da fadiga laríngea após sobrecarga vocal, averiguou que seu uso normalizou significativamente a combinação das medidas de limiar de pressão fonatória (PTP), incapacidade de produzir voz suave (IPSV) e frequência fundamental relativa (RFF), após aumento significativo ocasionado pela tarefa de sobrecarga vocal^(17)^. Em geral, os fonoaudiólogos brasileiros têm utilizado a FBM para alvos terapêuticos relacionados à função vocal, função musculoesquelética e função somatossensorial^(10)^.

No entanto, o debate sobre a indicação da FBM na modulação da inflamação ainda não alcançou um consenso, demonstrando a complexidade e a necessidade de uma análise mais profunda das evidências disponíveis e da prática clínica. A inflamação é um processo complexo e multifacetado, que pode ser parte de uma resposta protetora e de reparação tecidual, mas que, quando desregulada, também pode levar a danos e disfunções. A incerteza quanto ao uso da FBM para modulação da inflamação pode refletir a variabilidade dos resultados apresentados pelos estudos, uma possível heterogeneidade nas metodologias de pesquisa ou uma insuficiência de estudos de alta qualidade que forneçam evidências conclusivas. A discordância entre os especialistas pode ser um indicativo de que a experiência clínica e as expectativas dos pacientes não estão alinhadas com os dados de pesquisa ou que a interpretação desses dados varia significativamente entre os profissionais.

Além disso, a decisão de aplicar ou não a FBM no contexto da inflamação vocal pode ser influenciada por uma série de fatores, incluindo o tipo de disfonia, a etiologia da inflamação, o estágio da lesão laríngea e as condições específicas do paciente. A complexidade desses fatores pode dificultar a obtenção de um consenso sobre a aplicabilidade generalizada da FBM para todos os casos de processos inflamatórios nas pregas vocais.

### Momento da irradiação

Cinco itens foram objeto de consenso a respeito do momento da irradiação (itens 2, 22, 24, 48 e 51). Os consensos se referem à aplicação no início do processo de reabilitação vocal e antes e após a execução dos exercícios vocais em diferentes situações, na região laríngea, com diversos clientes/pacientes, incluídos profissionais da voz. Não houve consenso quanto ao uso da FBM durante e após os exercícios para condicionamento vocal (itens 23, 49 e 50).

O consenso alcançado pelos especialistas sobre o momento da irradiação (no início da reabilitação vocal, bem como antes e após a execução dos exercícios vocais) pode refletir uma tendência em reconhecer seus benefícios potenciais no preparo e recuperação da a musculatura envolvida na produção da voz. A decisão de aplicar a FBM no início da terapia pode estar ancorada na hipótese de que a preparação do tecido laríngeo poderia aumentar a eficácia dos exercícios vocais subsequentes. Ao melhorar a oxigenação e a circulação local, a FBM pode potencializar a resposta muscular, promovendo um estado mais receptivo para o treinamento e, consequentemente, otimizando os resultados da reabilitação. Além disso, ao aplicar a FBM antes e depois dos exercícios vocais em diferentes situações, os especialistas podem estar considerando a capacidade desse dispositivo em reduzir os sintomas de fadiga muscular e promover a recuperação. Um levantamento^(10)^ feito entre fonoaudiólogos brasileiros, demonstrou que eles tendem a usar a FBM antes da execução dos exercícios vocais, o que está em consonância com os achados do presente consenso.

A falta de consenso sobre a aplicação da FBM durante e após os exercícios vocais, no entanto, revela uma área de incerteza e debate. A hesitação em recomendar a FBM concomitantemente aos exercícios pode surgir de preocupações com a possibilidade de sobreestimulação dos tecidos laríngeos, o que poderia, teoricamente, levar a efeitos adversos ou diminuir a efetividade dos exercícios. Por outro lado, a movimentação no arcabouço laríngeo ocasionado pela maioria dos exercícios vocais, poderia reduzir a precisão do ponto de irradiação escolhido pelo clínico. A ausência de um acordo também pode ser atribuída à limitação de pesquisas que examinem os efeitos imediatos da FBM sobre os tecidos laríngeos em atividade.

Com relação à aplicação da FBM após os exercícios vocais pode ser uma área de divergência devido à complexidade das respostas fisiológicas envolvidas na recuperação muscular. Um estudo^(17)^ demonstrou os efeitos metabólicos e fotoquímicos da FBM nas pregas vocais promovendo uma fonação adequada com melhora nas medidas acústicas, aerodinâmicas e perceptivo-auditivas, além de auxiliar na recuperação muscular após o esforço, favorecendo o aporte energético para um equilíbrio muscular após os exercícios vocais. No entanto, os especialistas podem estar em busca de mais evidências que demonstrem a eficácia da FBM nesta fase, assegurando que seu uso não apenas é seguro, mas efetivamente contribui para a melhoria da função vocal.

Este panorama sugere a necessidade de mais pesquisas que abordem especificamente o *timing* da aplicação da FBM em relação aos exercícios vocais, bem como estudos que expliquem os mecanismos subjacentes aos seus efeitos na musculatura laríngea. Enquanto isso, a prática clínica deve ser conduzida por uma combinação de evidências científicas, experiência clínica e a individualidade das necessidades dos pacientes, sempre com uma abordagem conservadora no que diz respeito à segurança do paciente e à eficácia do tratamento.

### Dose de FBM realizada

Houve consenso entre os especialistas para doses a partir de 4J por ponto de irradiação (Itens 29, 30, 31, 41, 42). Os especialistas entraram em consenso para a dose de 6J para cada ponto de irradiação para tratamento de processos inflamatórios e 9J por ponto de irradiação para condicionamento vocal. Não houve consenso para doses menores que 4J por cada ponto de irradiação (Item 40).

Os achados referentes ao consenso entre os especialistas sobre as doses de FBM como parte da terapia vocal associada a processos inflamatórios e para condicionamento vocal são indicativos de um esforço em padronizar a aplicação desse dispositivo na área de voz. A concordância em doses mínimas de 4J por ponto de irradiação e a especificação de doses maiores para diferentes objetivos terapêuticos refletem uma orientação baseada tanto na experiência clínica, quanto em evidências emergentes de outras áreas da saúde.

A decisão de adotar uma dose de 4J ou 6J por ponto de irradiação para processos inflamatórios e 9J para condicionamento vocal sugere um reconhecimento dos especialistas sobre a necessidade de doses suficientemente potentes para alcançar os efeitos terapêuticos desejados. A dose de 6J pode ter sido escolhida como um ponto de equilíbrio que busca otimizar os efeitos anti-inflamatórios e analgésicos da FBM, fundamentando-se na evidência de que doses menores podem estimular a homeostase mitocondrial e acelerar a cicatrização tecidual^(24,95)^.

Por outro lado, a dose de 9J por ponto de irradiação para condicionamento vocal pode estar relacionada aos efeitos da FBM sobre as vias bioenergéticas e a modulação enzimática das fibras musculares. A eficácia da FBM, nesse contexto, está intrinsecamente ligada à sua capacidade de penetrar nas camadas musculares e induzir respostas bioquímicas benéficas. Doses mais elevadas podem ser necessárias para atingir as camadas mais profundas do tecido muscular, promovendo assim estímulos bioquímicos que aprimoram o desempenho muscular e aumentam a resistência à fadiga, aspectos cruciais para o condicionamento vocal, especialmente entre profissionais da voz^(96,97)^.o que é essencial para o condicionamento vocal, especialmente em profissionais da voz. Nesse sentido, é fundamental considerar a relação entre o comprimento de onda utilizado na FBM e seu poder de penetração no tecido, uma vez que ambos os fatores determinam a dosagem ótima necessária para alcançar efeitos terapêuticos desejados. O ajuste preciso do comprimento de onda pode otimizar a penetração da luz no tecido muscular, sugerindo que uma abordagem mais detalhada na seleção da dosagem pode potencializar os benefícios da FBM no condicionamento vocal^(96)^. Esta reflexão sobre a interdependência entre comprimento de onda, poder de penetração e dosagem é crucial para fundamentar nossa hipótese com uma base biológica e com o melhor conhecimento científico disponível.

Na presente pesquisa, os especialistas não referiram, na primeira rodada de entrevistas, o uso de doses menores que 4J no processo de terapia vocal de pacientes disfônicos, de modo que essa dosagem não entrou como item de julgamento na análise do consenso. Isso talvez reflita uma compreensão de que doses subterapêuticas podem não ser suficientes para induzir os efeitos biológicos necessários nos tecidos laríngeos, seja na terapia ou no treinamento vocal. Isso não exclui a possibilidade de que doses menores possam ter alguma utilidade em contextos específicos; contudo, os especialistas parecem inclinar-se para um limiar de eficácia que garanta uma resposta clínica perceptível.

A interpretação desses achados requer uma visão integrada que considere os mecanismos biológicos subjacentes à inflamação e à fisiologia vocal, bem como a resposta do tecido à irradiação. A padronização das doses de FBM representa um passo importante no desenvolvimento de protocolos de tratamento mais eficazes e seguros para a reabilitação vocal. No entanto, é crucial que essas recomendações sejam continuamente reavaliadas à luz de novas evidências científicas e da prática clínica reflexiva.

O presente consenso, embora embasado na experiência clínica e na transposição de achados de áreas afins, destaca a necessidade de ensaios clínicos futuros que confirmem a eficácia e a segurança das doses estipuladas. Tais estudos devem ser rigorosos, controlados e replicáveis, para que a comunidade científica possa confiar plenamente nas recomendações de dosagem e para que os clínicos possam aplicar a FBM com a maior precisão possível em seus tratamentos.

A aparente discrepância nos resultados da pesquisa, onde não se observou consenso entre os especialistas sobre o uso da FBM para a modulação da inflamação nas pregas vocais, em contraste com um consenso mais amplo sobre as doses específicas a partir de 4J por ponto de irradiação e o uso do comprimento de onda infravermelho para pacientes com processos inflamatórios, pode inicialmente parecer paradoxal. No entanto, uma análise mais detalhada desses achados pode revelar uma lógica subjacente que elucida essa aparente contradição.

Primeiramente, a falta de consenso sobre a indicação da FBM para modulação da inflamação pode refletir uma cautela geral dos especialistas quanto à aplicabilidade universal da FBM para todos os casos de inflamação nas pregas vocais. Essa cautela pode ser atribuída à complexidade dos processos inflamatórios, que podem variar significativamente em gravidade, etiologia e resposta ao tratamento. Além disso, a heterogeneidade nas condições clínicas dos pacientes e nas características específicas da inflamação pode levar os especialistas a adotarem uma abordagem mais individualizada, considerando a FBM como uma opção de tratamento entre várias outras, dependendo do contexto clínico específico de cada paciente.

Por outro lado, o consenso mais amplo sobre as doses específicas e o comprimento de onda infravermelho sugere que, uma vez que a decisão de utilizar a FBM é tomada, os especialistas têm uma possível preferência por determinadas doses de irradiação. Isso indica que, para os casos em que a FBM é considerada apropriada, existe uma forte concordância sobre como ela deve ser aplicada para maximizar a eficácia e minimizar os riscos. Portanto, a aparente discrepância nos resultados reflete uma abordagem pragmática e baseada em hipóteses sobre os efeitos da dose utilizada por parte dos especialistas.

### Local de aplicação

Quanto à localização da aplicação da FBM em região laríngea, os especialistas obtiveram consenso quanto à aplicação da FBM na lâmina da cartilagem tireóidea, na comissura anterior e na quilha da cartilagem tireóidea, tanto para situações de processo inflamatório nas pregas vocais, quanto para as situações de condicionamento vocal de profissionais da voz (Itens 27, 28, 52 e 53).

A escolha dessas áreas específicas para a aplicação da FBM é fundamentada na anatomia das pregas vocais e na sua relevância para a fisiologia vocal^(98)^. A lâmina da cartilagem tireóidea e a comissura anterior são pontos anatômicos cruciais, que oferecem um acesso mais direto às pregas vocais^(99,100)^ e, consequentemente, aos tecidos que podem se beneficiar dos efeitos da FBM. Anatomicamente, as pregas vocais se estendem horizontalmente na laringe, com corpo percorrendo paralelamente toda extensão das lâminas tireóideas, apresentando fixação anterior na face interna da cartilagem tireóidea, (formando a comissura anterior), e fixação posterior nos processos vocais e musculares das cartilagens aritenoides^(101,102)^. As pregas vocais são constituídas histologicamente (médio-lateralmente) por cinco camadas: epitélio estratificado, camadas superficial, intermediária e profunda da lâmina própria, músculo tireoaritenóideo^(103)^.

É importante notar que, para que a FBM seja eficaz, é necessário que a irradiação atinja toda a extensão da região-alvo. Isso é crucial porque as pregas vocais são constituídas por diferentes camadas que trabalham em conjunto para produzir a voz^(102)^. A irradiação precisa penetrar essas camadas de maneira adequada, para promover um efeito biomodulador, que possa influenciar positivamente o metabolismo celular, a redução da inflamação e a recuperação tecidual^(81,104)^.

O músculo tireoaritenóideo, que constitui o corpo das pregas vocais, é particularmente relevante na discussão sobre a aplicação da FBM^(99)^. Hipoteticamente, esse é o primeiro músculo intrínseco a receber a irradiação luminosa, devido à sua posição lateral. A eficácia da FBM na melhoria da função deste músculo pode ter implicações significativas no processo de produção vocal. A estimulação deste músculo pode ajudar na redução da tensão muscular^(98,99,105)^, o que é essencial para a recuperação de processos inflamatórios e para o condicionamento vocal, especialmente em profissionais da voz que dependem da resistência e flexibilidade deste músculo para um desempenho ótimo.

A concordância entre os especialistas sobre os locais de aplicação da FBM também demonstra o reconhecimento da importância de seguir parâmetros de irradiação bem estabelecidos. Fatores como o alvo, o método de aplicação, o comprimento de onda, a dose, a potência, a densidade de potência, o tipo de emissão, a densidade de energia, a energia total e o tempo são todos críticos para garantir que o tratamento seja não apenas eficaz, mas também seguro para o paciente^(89)^.

Em resumo, o consenso entre os especialistas sobre a localização da aplicação da FBM na região laríngea baseia-se na compreensão profunda da anatomia e fisiologia das pregas vocais, bem como na necessidade de direcionar a terapia para maximizar os benefícios enquanto minimiza os riscos. À medida que a pesquisa avance, é provável que mais refinamentos nos parâmetros de tratamento da FBM sejam estabelecidos, contribuindo para uma prática clínica ainda mais eficaz e informada.

### Modo e pontos de aplicação da FBM

Em relação às técnicas de aplicação da FBM, houve consenso no uso do contato pontual como método de irradiação em pacientes com processos inflamatórios nas pregas vocais e quando o objetivo terapêutico é melhorar o condicionamento vocal (itens 26, 44 e 51). Os especialistas obtiveram consenso para a aplicação em 3 e/ou 4 pontos, nas hemilaringes, quando o objetivo é melhorar o condicionamento vocal do cliente/paciente (itens 45, 46). Não houve consenso quanto à aplicação de 3-5 pontos nas hemilaringes de pacientes com processos inflamatórios nas pregas vocais, nem quanto à aplicação em cinco pontos nas hemilaringes de clientes cujo objetivo é melhorar o condicionamento vocal (itens 32-34 e 47).

O consenso em torno do uso da técnica de contato pontual como método de irradiação destaca a importância da precisão na aplicação da FBM, bem como a necessidade de uma abordagem direcionada que leve em consideração as peculiaridades anatômicas e funcionais da região laríngea^(100,101,106,107)^. O contato pontual foi consensual entre os especialistas devido à sua capacidade de minimizar a dispersão e a perda de energia, garantindo que a dose de irradiação seja entregue de forma mais eficiente ao tecido-alvo. Esta técnica é apontada como a mais adequada para alcançar as estruturas laríngeas, em particular a estrutura das pregas vocais e a musculatura intrínseca, que são cruciais para a produção da voz^(17,81)^. A literatura afim aborda que o contato pontual com ângulo de incidência de 90^o^ perpendicular ao alvo é o método mais adequado por diminuir a possibilidade de perda de energia por meio da reflexão^(81)^. Provavelmente, os especialistas em voz seguem esta recomendação com base nesses estudos.

A decisão de aplicar a FBM em 3 ou 4 pontos específicos nas hemilaringes para melhorar o condicionamento vocal pode refletir uma abordagem estratégica para cobrir as áreas mais relevantes para o funcionamento vocal, sem sobrecarregar a região com pontos de irradiação desnecessários.

A falta de consenso sobre a aplicação em 3-5 pontos para processos inflamatórios e em cinco pontos para o condicionamento vocal pode ser atribuída a uma série de fatores. Por um lado, pode haver uma consideração de que uma maior quantidade de pontos de irradiação possa não proporcionar benefícios adicionais significativos e que uma abordagem mais concentrada possa ser suficiente para promover os efeitos desejados. Por outro lado, o debate pode estar ligado a preocupações com a sobre-exposição e o potencial de efeitos adversos, especialmente em tecidos já comprometidos por processos inflamatórios.

É importante notar que a precisão na aplicação da FBM é crucial para garantir os efeitos terapêuticos desejados. O posicionamento impreciso dos pontos de irradiação pode não apenas reduzir a eficácia do tratamento, mas também aumentar o risco de efeitos indesejados^(92)^. A região laríngea é complexa^(100,107)^ e a precisão na aplicação da FBM é fundamental para estimular adequadamente as estruturas visadas sem afetar os tecidos circundantes.

Além disso, os parâmetros de aplicação, incluindo o número de pontos e a técnica de aplicação, devem ser cuidadosamente escolhidos para refletir os objetivos terapêuticos específicos, seja para reduzir a inflamação ou para melhorar o condicionamento vocal. A capacidade de penetração e os efeitos benéficos imediatos da FBM são aspectos críticos que podem efetivar o processo terapêutico e, portanto, devem ser considerados no delineamento do plano de tratamento^(108)^.

Em resumo, o consenso sobre a técnica de aplicação da FBM na região laríngea indica uma abordagem cuidadosa e metódica, que tem em vista tanto as características dos pacientes quanto as particularidades anatômicas e funcionais das pregas vocais. A discussão em torno do número ideal de pontos de irradiação reflete uma área de investigação contínua, que requer mais pesquisas para otimizar os protocolos de tratamento. A colaboração contínua entre pesquisa e prática clínica é essencial para refinar as técnicas de FBM, garantir os melhores resultados possíveis para os pacientes e avançar na prática baseada em evidências no campo da fonoaudiologia e terapia vocal.

### Comprimento de onda utilizado

Os especialistas obtiveram consenso quanto à utilização do comprimento de onda infravermelho em região laríngea, tanto em pacientes com processos inflamatórios nas pregas vocais, quanto em indivíduos cujo objetivo é a melhora do condicionamento vocal (Itens 25 e 43).

A escolha do comprimento de onda infravermelho para aplicações na região laríngea, tanto para processos inflamatórios nas pregas vocais quanto para a melhoria do condicionamento vocal, está fundamentada na capacidade de penetração mais profunda desse espectro luminoso^(93,94,109)^. A luz infravermelha é capaz de penetrar além das barreiras superficiais da pele, alcançando tecidos mais profundos como a musculatura intrínseca da laringe e as camadas das pregas vocais^(109)^.

Nos casos de processos inflamatórios, a utilização do infravermelho visa promover um efeito anti-inflamatório e analgésico em profundidade, aliviando os sintomas e acelerando o processo de cura. Dada a capacidade da luz infravermelha de atingir a derme e tecidos subjacentes^(24,110,111)^, os especialistas pode ter reconhecido seu potencial coadjuvante para modular processos inflamatórios nas pregas vocais.

Para o condicionamento vocal, principalmente em profissionais da voz, a escolha da luz infravermelha pode ser atribuída ao seu potencial de estimular o metabolismo celular e promover uma recuperação mais rápida de tecidos submetidos a uso constante e intensivo^(109)^. A energia fornecida pela luz infravermelha pode ajudar a otimizar a performance muscular, aumentando a resistência e a capacidade das pregas vocais de suportar o estresse vocal recorrente.

A aplicação de FBM com luz infravermelha na região laríngea, portanto, reflete um entendimento da interação complexa entre a luz e os tecidos biológicos, bem como uma apreciação dos mecanismos pelos quais a FBM pode oferecer benefícios terapêuticos. O consenso entre os especialistas realça a importância de uma abordagem no conhecimento científico disponível, que reconhece a 'janela óptica' do tecido humano como um determinante crucial para a eficácia da intervenção^(93,94,109)^.

Contudo, é importante ressaltar que o efeito da FBM não depende apenas do comprimento de onda, mas também de uma série de parâmetros ópticos cuidadosamente selecionados, como fluência, densidade de potência e estrutura de pulso^(112,113)^. É a combinação desses parâmetros que determinará a dose-resposta ótima e evitará efeitos subterapêuticos ou potencialmente prejudiciais.

Em resumo, o consenso sobre o uso do comprimento de onda infravermelho na prática fonoaudiológica para tratamento laríngeo evidencia a integração de conhecimentos avançados da interação luz-tecido e uma aplicação clínica inovadora que busca otimizar resultados terapêuticos tanto para patologias vocais quanto para o condicionamento de indivíduos saudáveis. Este consenso também serve como um impulso para a realização de pesquisas adicionais que possam confirmar ou refutar as hipóteses existentes, solidificando ainda mais a base científica da FBM na área de voz.

### Medidas de efeito após o uso da FBM

Para verificar o efeito da FBM na voz, especialistas solicitam uma tarefa vocal específica antes e após aplicação, assim como o relato do paciente/cliente (itens 35 e 36). Não houve concordância a respeito do uso da análise acústica para o mesmo fim (item 37).

A compreensão de intervenções com o uso da FBM na terapia vocal, envolve a utilização de métodos de avaliação que possam fornecer dados concretos sobre os efeitos terapêuticos desse dispositivo. O consenso entre especialistas quanto à utilização de tarefas vocais específicas e ao relato subjetivo do paciente/cliente antes e após a aplicação da FBM na voz reflete o reconhecimento da necessidade de uma avaliação funcional e experiencial para medir os resultados terapêuticos.

A solicitação de uma tarefa vocal específica antes e após (o que também é amplamente utilizado sob a expressão “prova terapêutica”) a aplicação da FBM permite uma comparação direta da função vocal^(7,114)^. Tais tarefas podem incluir a produção de sons sustentados, escalas vocais, ou frases faladas ou cantadas, que visam avaliar a qualidade vocal, a extensão vocal, a capacidade de projeção da voz, e outros parâmetros funcionais. Este método de avaliação é crucial para entender como a FBM pode afetar a biomecânica e aerodinâmica da produção da voz e proporcionar uma melhoria imediata e perceptível na função vocal^(115)^.

Além disso, o relato do paciente/cliente é igualmente importante, pois fornece uma dimensão subjetiva do impacto da FBM. A percepção do indivíduo sobre as mudanças na sua voz, conforto ao falar ou cantar, e a presença de sintomas como dor ou fadiga vocal são aspectos fundamentais que não podem ser captados apenas por medidas objetivas^(114,116-118)^. Esses relatos subjetivos são essenciais para uma compreensão holística dos efeitos da FBM no paciente.

No entanto, a falta de concordância sobre o uso da análise acústica pode estar relacionada a várias questões, tais como a acessibilidade e a complexidade dos equipamentos necessários, a variabilidade nas medidas devido a diferenças individuais ou a condições ambientais, ou até mesmo à falta de um consenso sobre quais parâmetros acústicos são os mais relevantes para a avaliação da eficácia da FBM^(115,119)^. A análise acústica da voz fornece dados objetivos sobre as características sonoras da voz, sendo uma ferramenta valiosa na pesquisa vocal e na prática clínica, pois pode detectar mudanças sutis na voz que podem não ser perceptíveis ao ouvido humano ou ao paciente^(115)^.

Uma revisão de escopo que analisou as medidas de avaliação vocal mais utilizadas para avaliação do efeito de treinamento em vozes saudáveis, identificou a análise acústica como a primeira opção e a autoavaliação como a terceira mais utilizada pelos fonoaudiólogos especialistas em voz^(120)^. Dessa forma, estudos futuros precisam definir quais os desfechos clínicos principais a serem considerados para avaliação da eficácia da FBM na área de voz.

## LIMITAÇÕES E DIRECIONAMENTOS PARA FUTURAS PESQUISAS

Este estudo de consenso traz contribuições importantes para a fonoaudiologia, especialmente no uso da FBM na terapia e treinamento vocal, marcando um avanço na padronização de procedimentos clínicos e reforçando a prática baseada em evidências. A integração da opinião de especialistas e das preferências dos pacientes, mesmo na ausência de evidências robustas, alinha-se ao modelo tripartido da prática baseada em evidências, que valoriza a evidência externa, a experiência clínica e as necessidades dos pacientes. A complexidade crescente das demandas clínicas exige que a prática e a pesquisa evoluam juntas, sugerindo a necessidade de futuros estudos focados em expandir o corpo de evidências externas por meio de pesquisas controladas e randomizadas^(37,45,121)^.

A falta de evidências científicas robustas sobre a FBM na terapia vocal implica uma maior dependência do conhecimento especializado dos fonoaudiólogos para adaptar o tratamento às necessidades individuais dos pacientes. A implementação da FBM deve ser guiada por uma compreensão dos mecanismos biológicos da voz e princípios bioéticos, enfatizando a transparência sobre os benefícios e riscos potenciais. Este estudo estabelece diretrizes importantes, mas reconhece a necessidade contínua de pesquisa baseada em evidências para refinar a aplicação da FBM na prática vocal.

As descrições geradas a partir deste estudo podem servir de suporte para que fonoaudiólogos clínicos que trabalham com a habilitação e/ou reabilitação vocal obtenham uma normativa de como este dispositivo vem sendo utilizado por especialistas na área. Também serve aos fonoaudiólogos pesquisadores, para que transformem os itens deste consenso em hipóteses de pesquisa, a fim de que sejam testadas e comprovadas sua eficácia ou ineficácia.

Na implementação da FBM para terapia e treinamento vocal, clínicos devem navegar um campo com evidência científica limitada, enfatizando a compreensão dos mecanismos biológicos e fisiopatológicos da voz e aderindo estritamente a princípios bioéticos. A transparência com os pacientes quanto aos potenciais benefícios e riscos é essencial, assim como a garantia de que as dosagens de luz sejam seguras e eficazes. A beneficência é um objetivo chave, buscando promover a saúde vocal e a recuperação de lesões com o uso informado da FBM, enquanto a justiça se manifesta no acesso equitativo a essas terapias. O compromisso com a ética e o conhecimento científico permite aos clínicos uma prática responsável na reabilitação vocal com FBM, apesar das limitações evidentes na pesquisa atual.

Em suma, as diretrizes estabelecidas neste estudo são um marco na prática da fonoaudiologia no campo da voz, contudo, o campo deve continuar a buscar uma compreensão mais profunda e baseada em evidências sobre a aplicação da FBM, adaptando-se à medida que novas informações se tornam disponíveis.

## CONCLUSÃO

Houve consenso com relação à indicação da FBM como dispositivo complementar à terapia e ao treinamento vocal convencional. Os especialistas concordaram que a FBM pode ser utilizada em profissionais da voz sem disfonia e em pacientes disfônicos, especificamente em casos de disfonia comportamental sem lesão laríngea. Os alvos terapêuticos associados ao uso da FBM envolveram melhora do desempenho e recuperação muscular e diminuição dos sintomas de fadiga vocal do cliente/paciente, incluindo profissionais da voz. Os especialistas consideram que a FBM deve ser aplicada no início do processo de reabilitação, antes e após a execução de exercícios vocais. Os especialistas entraram em consenso para a dose de 4J e 6J para cada ponto de irradiação para tratamento de processos inflamatórios e 9J por ponto de irradiação para condicionamento vocal. Houve consenso quanto à aplicação da FBM na lâmina da cartilagem tireóidea, na comissura anterior e na quilha da cartilagem tireóidea, tanto para situações de processo inflamatório nas pregas vocais, quanto para as situações de condicionamento vocal de profissionais da voz. O uso do contato pontual foi consenso entre os especialistas, com aplicação em 3 e/ou 4 pontos, nas hemilaringes, quando o objetivo é melhorar o condicionamento vocal do cliente/paciente. Os especialistas obtiveram consenso quanto à utilização do comprimento de onda infravermelho em região laríngea, tanto em pacientes com processos inflamatórios nas pregas vocais, quanto em indivíduos cujo objetivo é a melhora do condicionamento vocal. Os especialistas concordaram com o uso de uma tarefa vocal pré e pós administração da FBM, assim como o relato do paciente/cliente para avaliar o efeito da FBM.
